# Facet‐Engineered ZnO as an Interfacial Regulator for Stable Lithium Metal Anodes

**DOI:** 10.1002/smll.202514924

**Published:** 2026-03-01

**Authors:** Kyungmin Kim, Seonghyun Park, Hwanju Lim, Jeongwoo Lee, Sohyung Jiong, Xinqi Chen, Dahyun Daniel Lim, Byungseok Seo, Wonjoon Choi

**Affiliations:** ^1^ School of Mechanical Engineering Korea University Seoul Republic of Korea; ^2^ Department of Mechanical Engineering Northwestern University Evanston Illinois USA; ^3^ The NUANCE Center Northwestern University Evanston Illinois USA

**Keywords:** interfacial engineering, Li plating/stripping, Li‐metal batteries, thermal processing, zinc oxides

## Abstract

Interfacial instability remains the primary obstacle to realizing high‐energy Li‐metal batteries (LMBs). Here, we demonstrate that ZnO can function not as a conventional anode material but as a facet‐engineered interfacial regulator that stabilizes Li‐metal deposition. Using electrothermal‐wave (ETW) processing, we precisely modulate atomic diffusion kinetics to tailor the crystal facet orientation and morphology of ZnO nanostructures on carbon fibers. This controllable platform enables decoupling the facet‐ and morphology‐dependent effects on interfacial stability. Mechanistic analyses reveal that the semipolar (101) facet forms a conductive Li–Zn interface that promotes uniform Li nucleation and enables long‐term plating/stripping stability (>800 h), whereas the polar (002) facet generates an insulating Li_2_O‐rich layer that impedes charge transfer. Concurrently, a 2D planar morphology enhances the electrochemically active surface area and exchange current density, yielding more favorable Li plating kinetics than 3D architectures. The optimized ZnO@CF electrode, integrating the (101)‐dominant facet and planar configuration, delivers stable reversibility in half‐cells and retains 80 % capacity over 200 cycles at 0.5 C in full‐cell pairing with a commercial NCM523 cathode. This study establishes a design framework where facet‐dependent interfacial reactivity and morphology‐dependent kinetic regulation act synergistically to stabilize reactive metal interfaces, offering a new paradigm for durable and efficient LMB anodes.

## Graphical abstract

Programmable electrothermal‐wave (ETW) processing enables precise facet and morphology control of ZnO, redefining it as an interfacial regulator for stabilizing Li‐metal anodes. ETW‐controlled crystallization modulates diffusion pathways to produce facet‐specific and morphology‐defined ZnO that governs Li nucleation. The semipolar (101) facet forms a conductive Li–Zn interface for uniform Li deposition, whereas the polar (002) facet promotes an insulating Li_2_O layer and instability. Planar 2D structures further provide kinetically favorable, spatially uniform nucleation sites compared to 3D architectures. This synergistic facet–morphology design enables stable Li plating/stripping and improved reversibility in Li‐metal batteries.

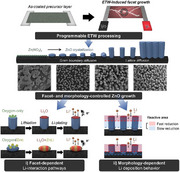

## 1 Introduction

Interfacial instability remains the primary bottleneck preventing the practical deployment of high‐energy Li‐metal batteries (LMBs) [1, 2]. Uncontrolled Li nucleation, dendritic growth, and continuous interphase reconstruction cause rapid capacity fading and severe safety hazards [3, 4, 5]. Although electrolyte optimization and surface protective coatings have partially resolved these issues, such approaches largely mitigate the symptoms rather than addressing the fundamental cause, i.e., the inherent instability of the Li–electrolyte interface during repeated plating and stripping [6, 7]. In this context, the interfacial configuration of the electrode itself plays a crucial role in governing overall stability. Accordingly, considerable research has focused on exploring electrode architectures that incorporate lithiophilic materials to regulate Li affinity, promote homogeneous nucleation, and suppress dendrite growth [8]. Among these interfacial design strategies, metal oxides have emerged as promising regulators capable of forming chemically stable, mechanically robust, and ionically compatible Li–electrode interfaces [4, 9, 10, 11].

Within this framework, metal oxides have been extensively exploited as interfacial regulators and active components in electrochemical systems such as electrocatalysts and battery electrodes owing to their structural versatility, redox activity, and chemical robustness [12, 13, 14, 15]. Their abundance and low cost further enhance their attractiveness for practical applications. Oxides such as TiO_2_, Al_2_O_3_, SnO_2_, and MgO have been used as protective or lithiophilic layers for Li anodes, forming ionically conductive yet mechanically resilient interphases [4, 9, 10].

Among these oxides, zinc oxide (ZnO), a representative wurtzite‐structured material, combines strong intrinsic lithiophilicity with high chemical stability and cost‐effectiveness [16, 17, 18]. Nevertheless, ZnO has historically received less attention than other anode materials such as silicon and graphite [19]. In conventional Li‐ion batteries (LIBs), ZnO has been investigated as an active anode material because of its high theoretical capacity and strong affinity for Li [2, 20, 21, 22]. However, its conversion‐type lithiation reaction typically causes large volume changes, severe interfacial instability, and sluggish reaction kinetics, leading to poor cycle retention and low rate performance. Moreover, the gradual evolution of its electrochemical potential reflects a continuous redox process rather than distinct phase transitions, unlike alloying or intercalation anodes (e.g., Si, graphite) that exhibit step‐like potential plateaus. Consequently, oxide‐based anodes inherently suffer from broad voltage hysteresis, slower kinetics, and limited power density, making it difficult for ZnO to outperform advanced anode materials in conventional LIB configurations. Thus, despite its lithiophilicity and structural simplicity, ZnO has long been regarded as an unstable and low‐performing anode material.

Compared with those for LIBs, the design priorities for LMBs fundamentally differ; instead of maximizing the capacity, current research focuses on stabilizing Li deposition and suppressing dendrite formation [23, 24]. Under this paradigm, the lithiophilicity that enables continuous conversion reactions in LIBs is a decisive advantage for LMBs. The polar surfaces of ZnO facilitate homogeneous Li nucleation and serve as chemically compatible sites for metal deposition, thereby improving the reversibility of Li plating and stripping. In this regard, ZnO can be redefined not as a conventional capacity‐bearing oxide but as an interfacial regulator that promotes uniform Li deposition and stabilizes the Li–electrolyte interface.

Importantly, the efficacy of ZnO as an interfacial regulator is not solely determined by its bulk composition, but also by its crystal orientation and surface termination. Owing to its polar wurtzite structure, ZnO exhibits distinct interfacial properties depending on its exposed facet; specifically, the polarity and atomic arrangement dictate the surface energy, charge distribution, and Li adsorption characteristics [25, 26, 27, 28]. These parameters strongly influence the Li adsorption energetics, nucleation overpotentials, and charge transfer behavior during metal plating. Such facet‐dependent interfacial properties mirror trends observed in catalysis and electrode surface science, where subtle variations in exposed facets dictate reaction pathways and interfacial stability [25, 26, 29]. Extending these insights to LMBs offers a rational route to controlling ion transport and nucleation dynamics at the nanoscale. Hence, elucidating how morphology‐ and facet‐dependent features govern Li‐metal interfacial behavior is essential for designing durable LMB anodes.

Despite the significance of these structural factors, conventional crystal manipulation techniques, such as hydrothermal synthesis and vapor deposition, often face challenges in continuously tuning morphological and crystallographic characteristics [30, 31]. Because these methods typically rely on slow reaction kinetics under near‐equilibrium conditions, they inherently favor the formation of thermodynamically stable phases, often bypassing unique metastable states that could harbor superior interfacial properties [32]. This limitation hinders the systematic decoupling of facet‐ and morphology‐dependent effects, as the ability to stabilize unconventional nanostructures remains restricted within equilibrium boundaries. Consequently, there is a critical need for a synthesis platform capable of operating in a non‐equilibrium regime to explore and access these hidden structural configurations [33].

Herein, we introduce electrothermal wave (ETW) processing as a programmable thermal modulation technique that continuously tailors the exposed facet and morphology of ZnO directly from precursors. ETW generates sub‐second temperature pulses, enabling fine‐tuning of diffusion kinetics during crystal growth. By adjusting the ETW temperature profile, the dominant diffusion pathway, either lattice‐ or grain‐boundary‐driven, can be selectively regulated, yielding distinct morphological evolution and facet‐defined surfaces in the resulting ZnO nanostructures. This controllable synthesis platform allows systematic decoupling of facet‐ and morphology‐dependent effects, providing direct insight into how each factor governs interfacial stability and Li plating behavior in ZnO‐based LMB anodes. Using this approach, we quantitatively evaluated the relative contributions of structural orientation and morphology to electrochemical stability and identified the optimal configuration for LMB operation. The optimized ZnO@carbon fiber (CF) electrode exhibited outstanding cycling durability, retaining ∼80 % capacity over 200 cycles at 0.5 C with an areal capacity of ∼2 mAh cm^−2^ when paired with a commercial LiNi_0.5_Co_0.2_Mn_0.3_O_2_ (NCM523) cathode.

## 2 Results and Discussion

### 2.1 Fabrication and Thermal Profiling of ZnO@CF Electrodes Under ETW Conditions

**FIGURE 1:**
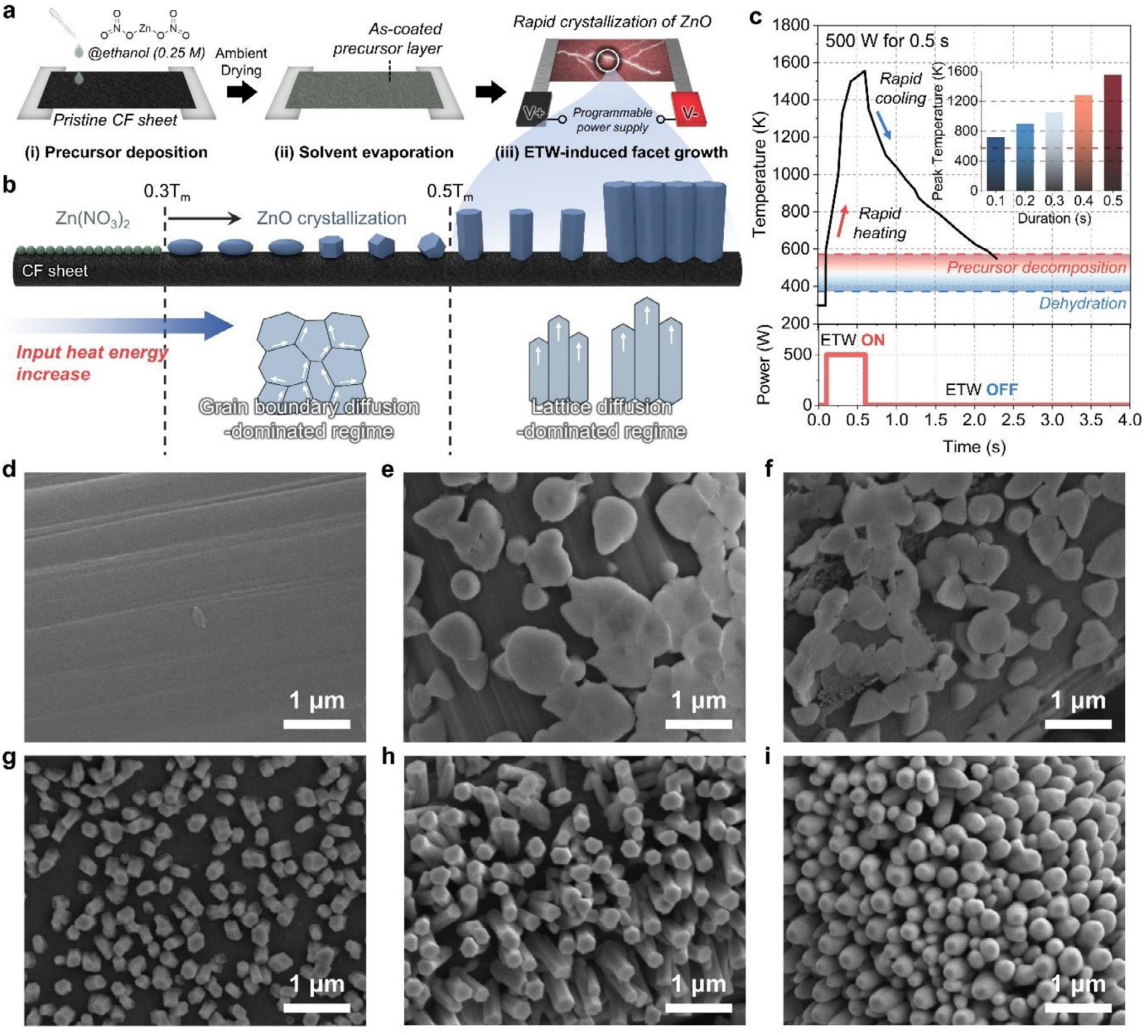
Preparation of ZnO@CF composites and structural evolution under different ETW conditions. (a) Schematic illustration of the fabrication process, including precursor deposition, solvent evaporation, and rapid ETW‐induced crystallization. (b) Conceptual diagram showing the transition from grain‐boundary–diffusion–dominated to lattice‐diffusion–dominated growth with increasing input heat energy. (c) Real‐time temperature profile for a representative ETW pulse (500 W), highlighting rapid heating, precursor decomposition, and subsequent cooling; inset shows the peak temperature as a function of pulse duration. (d–i) Scanning electron microscopy (SEM) images of (d) bare CF, (e) ZnO@CF‐0.1, (f) ZnO@CF‐0.2, (g) ZnO@CF‐0.3, (h) ZnO@CF‐0.4, and (i) ZnO@CF‐0.5, demonstrating the systematic morphological transition from planar films to vertically aligned ZnO nanostructures.

Figure 1a schematically illustrates the overall fabrication procedure used to prepare the ZnO@CF electrodes. CF sheets were cut into rectangular pieces (1 × 2 cm^2^), after which a zinc nitrate precursor solution (0.1 m in 75 % ethanol) was drop‐cast onto the pristine CF substrates. A mixed 75 % ethanol solvent was chosen to ensure uniform wetting and controlled evaporation, thereby promoting homogeneous precursor coverage and minimizing local concentration gradients during drying. The samples were then air‐dried for 24 h at room temperature to complete solvent removal and achieve uniform precursor deposition.

The subsequent ETW treatment was designed to exploit the temperature‐dependent kinetics of precursor decomposition, atomic diffusion, and crystal growth to realize controlled morphological and crystallographic evolution. Specifically, the pulse duration was varied to realize differential thermal modulation, enabling precise control over the facet orientation and morphology of the ZnO nanostructures while minimizing undesired thermal changes in the surface characteristics of the CF substrate. After pre‐screening the applicable energy window at which precursor decomposition occurs without inducing thermal damage to the CF substrate, the ETW input parameters were fixed at 25 A and 20 V. Under these conditions, pulse duration emerged as the dominant variable governing the ZnO growth pathway; therefore, only the pulse duration was varied to achieve differential thermal modulation while keeping electrical input constant. A single ETW pulse was applied to each sample for the designated durations (0.1–0.5 s), and the resulting electrodes are hereafter denoted as ZnO@CF‐x.

Figure 1b illustrates a schematic description of the thermally activated diffusion mechanisms governing ZnO crystal growth, constructed as a function of the absolute temperature (*T*_m_ = melting point, ∼2250 K). According to classical diffusion theory for metal oxides [34, 35], grain‐boundary (GB) diffusion dominates at lower normalized temperatures (<0.5*T*_m_), whereas lattice diffusion becomes prevalent beyond this threshold. Thus, the diagram delineates two distinct kinetic regimes corresponding to 0.3–0.5*T*_m_ (GB‐controlled growth) and >0.5*T*_m_ (lattice‐controlled growth).

Real‐time thermal profiles under each ETW condition are shown in Figure 1c and Figure S1. Evidently, the recorded temperature transients could confirm that varying the pulse duration effectively subjected the samples to different peak temperatures and exposure times, providing five distinct cases for studying diffusion‐driven growth. Specifically, ZnO@CF‐0.1 experienced only a brief excursion above the precursor decomposition temperature, resulting in limited nucleation. In contrast, ZnO@CF‐0.2 and ZnO@CF‐0.3 were processed entirely within the intermediate 0.3–0.5*T_m_* window, where GB diffusion dominates, but lattice diffusion begins to activate, promoting rapid grain coalescence. Notably, ZnO@CF‐0.3 reached a peak temperature close to the upper threshold of GB‐dominated behavior (∼0.5*T*_m_). In contrast, both ZnO@CF‐0.4 and ZnO@CF‐0.5 surpassed the 0.5 *T*_m_ limit, indicating that lattice diffusion was likely the primary mechanism governing crystal growth and morphological evolution in these samples. These findings demonstrate that ETW modulation provides a reproducible and deterministic means of controlling the dominant diffusion mechanism that governs ZnO nanostructure formation. By selectively tuning the thermal trajectory through pulse duration, the ETW process enables precise regulation of crystallographic orientation and morphology on CF substrates, establishing a robust platform for mechanistically probing facet‐ and morphology‐dependent interfacial behavior in subsequent electrochemical analyses.

### 2.2 Morphological Evolution and Diffusion Mechanism Analysis

The ETW treatment induced a systematic and pulse‐duration‐dependent morphological transformation in the ZnO nanostructures, as verified by SEM. Figure 1d shows the pristine CF substrate as a reference, while Figure 1e–i display the corresponding surfaces of ZnO@CF electrodes fabricated under different pulse durations. The morphological transition exhibited a clear dependence on the applied pulse time, directly reflecting the dominant atomic diffusion mechanism under each condition. At short pulse durations (ZnO@CF‐0.1 and ZnO@CF‐0.2; Figure 1e,f), the temperature remained within the GB diffusion regime (*T* < 0.5*T*_m_). Under these conditions, the deposited ZnO preserved a nearly planar morphology reminiscent of the precursor film, suggesting minimal atomic rearrangement and restricted crystal growth. Nevertheless, a slight enhancement in local crystallinity was detected even for ZnO@CF‐0.2, as indicated by the reduced amorphous background and narrower X‐ray diffraction (XRD) peak widths (Table S1), indicating the onset of GB‐driven coalescence even before lattice diffusion becomes active.

At an intermediate duration of 0.3 s (ZnO@CF‐0.3; Figure 1g), the ETW‐driven reconstruction reached a critical transition between GB‐ and lattice‐diffusion‐dominated growth. At this threshold, ZnO began to crystallize into small, distinct cubic grains, although they remained randomly oriented, which was consistent with an annealing‐like process driven by enhanced atomic mobility. This interpretation is corroborated by the full width at half maximum (FWHM) of the XRD reflections from 0.1 to 0.3 s (Table S1). Specifically, the FWHM systematically decreased as the duration was prolonged from 0.1 to 0.3 s, quantitatively confirming that the local crystallinity improved even while GB diffusion remained dominant.

The significance of this threshold behavior is further supported by additional control experiments (Figure S2). Multiple ETW pulses at 0.2 s, remaining below the threshold temperature, produced negligible morphological change, whereas sequential pulses of 0.2 s followed by 0.3 s crossing the threshold successfully initiated crystallization. This finding clearly indicates that the instantaneous peak temperature reached at 0.3 s, rather than cumulative thermal exposure, is the decisive factor governing the activation of the lattice‐diffusion process. At longer pulse durations (ZnO@CF‐0.4 and ZnO@CF‐0.5; Figure 1h,i), the temperature exceeded 0.5*T*_m_, entering the lattice diffusion regime. The activation of bulk atomic transport under these conditions led to substantial morphological reorganization: the cubic ZnO grains not only coarsened but also preferentially aligned perpendicular to the CF substrate [28, 35]. Such anisotropic orientation is a recognized hallmark of lattice‐diffusion‐dominated growth, analogous to directional sintering, wherein mass transport along lattice planes drives oriented structural evolution.

Collectively, these observations demonstrate that the ETW processing enables mechanistic control over both morphology and crystallinity of ZnO through selective activation of temperature‐dependent atomic diffusion pathways. The ability to tune the transition between GB‐ and lattice‐diffusion regimes offers a versatile route for tailoring nanoscale architectures and surface anisotropy in oxide‐based interfacial materials.

### 2.3 Physicochemical Characterization of ZnO@CF Formed Under Different ETW Conditions

**FIGURE 2:**
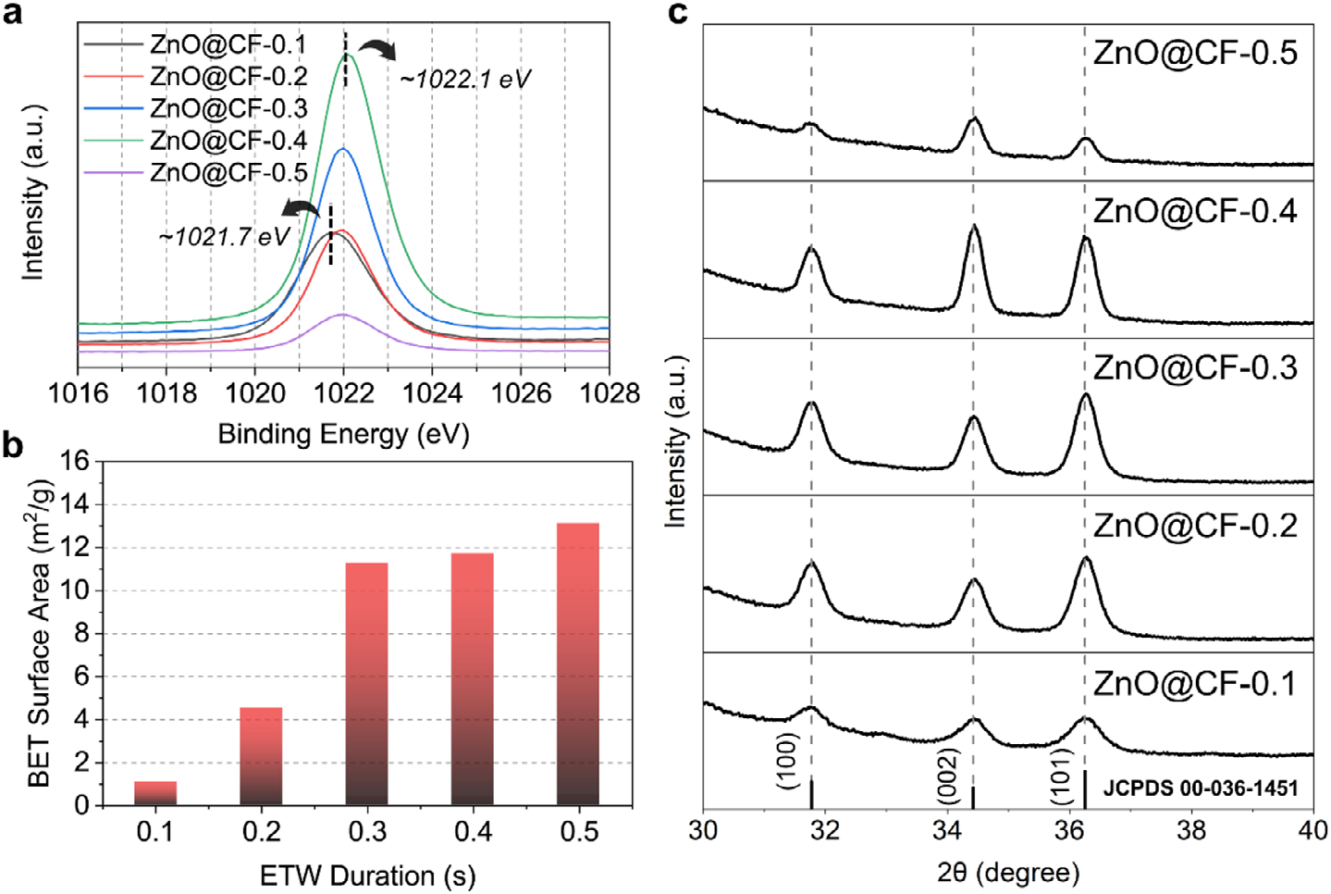
Physicochemical characterization of the ZnO@CF composites. (a) XPS spectra of the Zn 2p 3/2 core level, showing the shift toward higher binding energy (∼1022.1 eV), indicating the complete conversion of the precursor to stoichiometric ZnO with increasing ETW duration. (b) Specific surface area of the ZnO@CF samples obtained from Brunauer–Emmett–Teller (BET) analysis as a function of ETW duration. (c) XRD patterns presenting the evolution of the (100), (002), and (101) reflections of the ZnO@CF samples.

The surface morphology of ZnO@CF varied markedly with ETW pulse duration (Figure 1d–i). To establish a reliable baseline for comparison, the chemical composition of each sample was first analyzed. X‐ray photoelectron spectroscopy (XPS) analysis revealed a noticeable amount of nitrogen in ZnO@CF‐0.1, suggesting incomplete precursor decomposition (Table S2). This incomplete conversion was also reflected in the Zn 2p 3/2 core‐level spectra (Figure 2a; Figure S3). The peak for the ZnO@CF‐0.1 sample shifted to a lower binding energy (1021.7 eV), whereas all other samples (ZnO@CF‐0.2 through ZnO@CF‐0.5) showed peaks near 1022.0–1022.1 eV, consistent with stoichiometric ZnO [36, 37].

Conversely, complementary energy‐dispersive X‐ray spectroscopy (EDX, Figure S4) confirmed that ZnO@CF‐0.2 consisted solely of Zn and O, verifying that a 0.2 s ETW pulse was sufficient for complete chemical conversion. Owing to its incomplete reaction and distinct surface chemistry, ZnO@CF‐0.1 was excluded from subsequent electrochemical analyses.

BET measurements revealed an evolution of the surface area and pore size with pulse duration (Figure 2b; Figure S5). Specific surface area increased with pulse duration, showing a rapid increase up to 0.3 s, while further changes between 0.3 and 0.5 s were relatively minor. This aligns with the morphological evolution observed by SEM. The transition from a planar film (0.1 and 0.2 s) to discrete crystallites (0.3–0.5 s) demonstrates a pronounced increase in surface area. However, the substrates were also exposed to ETW in air at elevated temperature, which can generate surface defects and thereby increase the apparent surface area. At the same time, lattice‐diffusion‐driven coarsening tends to reduce the accessible area. Considering these effects together, the increase in specific surface area beyond 0.3 s is unlikely to reflect an actual increase in the ZnO surface area. The aspect ratio of the particles observed by SEM was close to 1 at 0.3 s, while it increased to ∼3 at 0.4 and 0.5 s. Assuming identical Zn loading across samples, the structures at 0.4 and 0.5 s are therefore expected to have a surface area that is reduced by no more than ∼20 % compared with the 0.3 s sample.

This structural evolution was closely coupled with a distinct change in crystallographic texture. As described in Section 2.2, ZnO@CF‐0.2 and ZnO@CF‐0.3, which formed within the grain‐boundary‐diffusion regime, displayed randomly oriented crystallites. In contrast, ZnO@CF‐0.4 and ZnO@CF‐0.5, which formed within the lattice‐diffusion regime, exhibited pronounced directional growth perpendicular to the substrate (Figure 1h), resulting in vertically aligned ZnO rods. This orientation evolution is directly reflected in the XRD patterns (Figure 2c). The (002) diffraction peak, corresponding to the polar *c*‐axis basal plane of wurtzite ZnO, became noticeably more intense at 0.4 s, while among the non‐polar side‐plane reflections, the (100) peak remained nearly constant and the (101) peak decreased. At 0.5 s, SEM revealed dense aggregation (Figure 1i) and the (002) peak further intensified, confirming the dominance of vertically oriented crystallites. In summary, the transition from randomly oriented grains (ZnO@CF‐0.2 and 0.3) to vertically aligned rods (ZnO@CF‐0.4 and 0.5) correlates directly with the enhancement of the (002) reflection and the attenuation of the (101) peak. Notably, wurtzite‐type ZnO exhibits three representative crystallographic planes—(100), (101), and (002)—each characterized by distinct atomic arrangements, surface energies, and electrochemical reactivities. Among them, the (002) plane corresponds to the polar *c*‐axis basal plane, whereas the (100) and (101) planes are non‐polar side facets. These three planes served as benchmarks for analyzing the evolution of facets in the ZnO nanostructures and were referenced throughout the morphological and XRD‐based analyses in this study. Furthermore, the presence and precise distribution of these crystallographic facets were cross‐verified at the nanoscale using transmission electron microscopy (TEM) and selected area electron diffraction (SAED) (Figure S6).

Interestingly, the dominance of the (002) orientation is thermodynamically unexpected, as this polar plane possesses a higher surface energy than the (100) or (101) planes under equilibrium conditions [16]. We attribute this non‐equilibrium preference to two primary factors. First, a geometric shielding effect occurs during vertical growth: the (002) facet corresponds to the top basal plane of the hexagonal crystal, whereas the (100) and (101) facets constitute the lateral surfaces. As neighboring rods aggregate laterally (Figure 1h,i), the (100) and (101) side facets become increasingly obscured and contribute less to the diffracted intensity, while the exposed top (002) facets dominate the XRD signal. Second, the kinetically driven nature of ETW promotes nonequilibrium stabilization of high‐energy surfaces. Rapid heating and quenching under oxidizing conditions can kinetically freeze the (002) orientation, which forms preferentially during rapid nucleation and crystal growth [38, 39, 40, 41]. Overall, these findings demonstrate that the ETW pulse duration serves as a decisive parameter for tailoring both the crystallographic texture and morphological evolution of ZnO nanostructures. ZnO@CF‐0.2 marked the threshold for complete precursor decomposition, ZnO@CF‐0.3 indicates the onset of crystallite formation, and ZnO@CF‐0.4 and ZnO@CF‐0.5 exhibit progressive facet reorientation and anisotropic growth dominated by the (002) orientation.

### 2.4 Mode‐Dependent Electrochemical Behavior and Mechanistic Correlation

**FIGURE 3:**
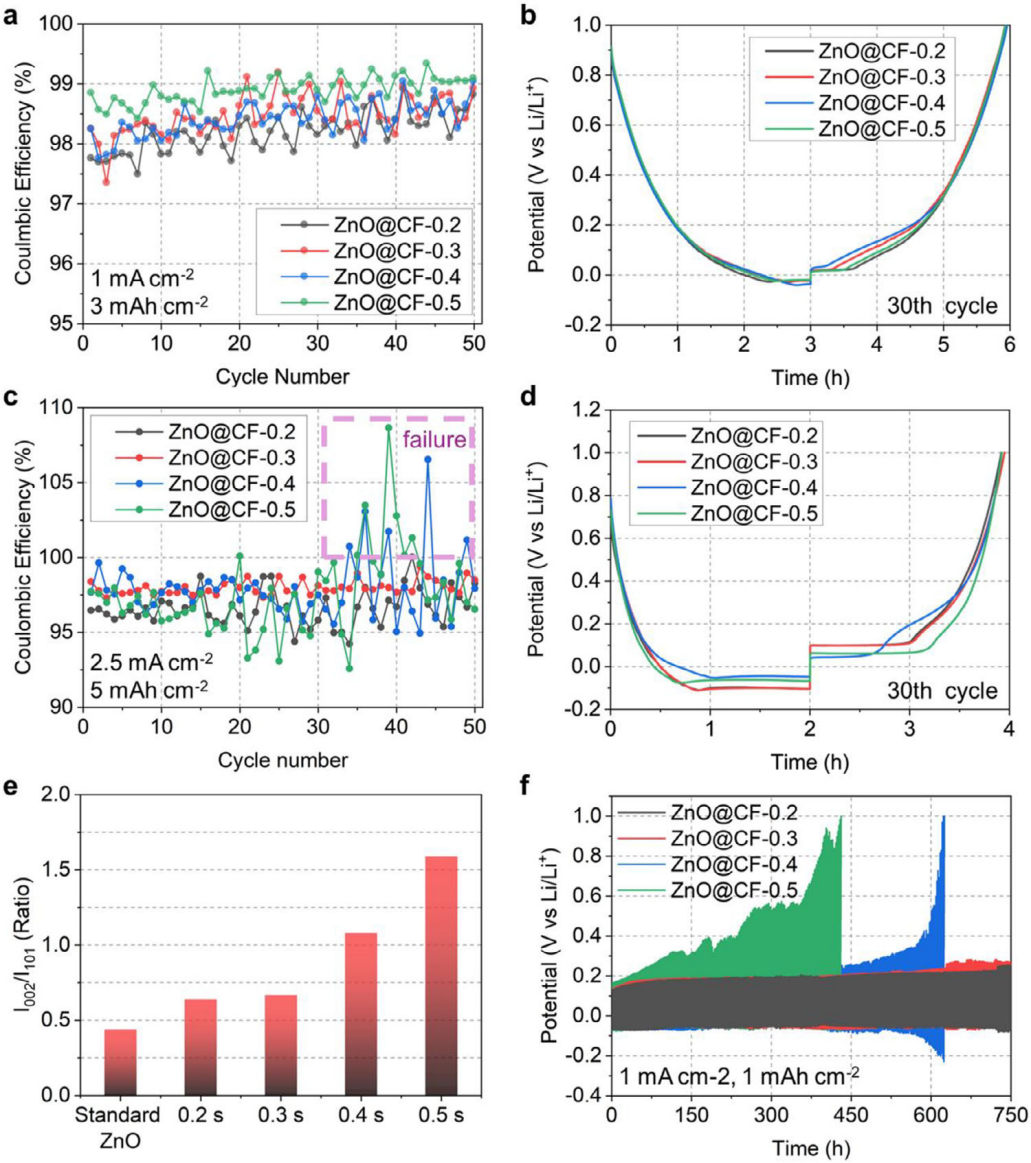
Electrochemical performance of the ZnO@CF electrodes under LIB‐ and LMB‐type operating conditions and correlation with facet exposure. (a) Coulombic efficiencies (CEs) of ZnO@CF‐0.2, ZnO@CF‐0.3, ZnO@CF‐0.4, and ZnO@CF‐0.5 in half‐cells operated in LIB mode, under a current density of 1 mA cm^−2^ with a capacity of 3 mAh cm^−2^. (b) Corresponding voltage profiles at the 30th cycle under LIB‐mode operation. (c) CEs of the same electrodes under LMB‐mode conditions (2.5 mA cm^−2^, 5 mAh cm^−2^). (d) Voltage profiles at the 30th cycle under LMB‐mode operation. (e) I_002_/I_101_ ratios extracted from XRD patterns, used to compare the relative exposure of the (002) and (101) facets. (f) Pseudo‐ symmetric‐cell cycling test of the Li plated ZnO@CF electrodes vs. the Li chip at 1 mA cm^−2^ and 1 mAh cm^−2^.

To evaluate the facet‐dependent reactivity of the ZnO@CF electrodes, we first established a working hypothesis based on differences in their atomic structures. The (002) and (101) facets were selected for analysis because their relative exposures exhibited the most pronounced evolution with ETW pulse duration. In wurtzite ZnO, the (002) facet is polar and consists of alternating Zn and O layers stacked along the *c*‐axis, resulting in basal surfaces terminated exclusively either by Zn or O. In contrast, the semipolar (101) facet exposes a mixed Zn–O atomic arrangement in each surface unit, rendering it non‐polar. These structural differences provide a rational basis for facet‐dependent variation in electrochemical reactivity [39, 42]. Galvanostatic cycling was performed under two operating modes to confirm the hypothesis (Figure 3a–d). In the LIB mode (referring to Li storage via conversion and intercalation reactions), half‐cells were cycled at 1 mA cm^−2^ (capacity limit 3 mAh cm^−2^) (Figure 3a,b). In the LMB mode (referring to Li storage via metallic Li plating and stripping), the current density was increased to 2.5 mA cm^−2^ (5 mAh cm^−2^) to accentuate kinetic contrasts associated with Li metal deposition (Figure 3c,d).

The two operating modes exhibited distinctly different trends. In the LIB mode, the CEs for ZnO@CF‐0.2, ZnO@CF‐0.3, ZnO@CF‐0.4, and ZnO@CF‐0.5 over 50 cycles were approximately 98.7 %, 98.8 %, 99.1 %, and 99.2 %, respectively, with corresponding average CEs of 98.2 %, 98.4 %, 98.5 %, and 98.9 %. By contrast, in the LMB mode, the corresponding CEs after 50 cycles were approximately 98.2 %, 98.5 %, 97.4 %, and 96.5 %, with average values of 97.0 %, 97.9 %, 97.7 %, and 97.4 %. While ZnO@CF‐0.2 and ‐0.3 remained relatively stable, ZnO@CF‐0.4 and ‐0.5 displayed pronounced CE fluctuations (∼110 %) as they approached the end of their cycle life, indicating a significant loss of electrochemical reliability. When excluding these unstable failure regions, ZnO@CF‐0.4 and ‐0.5 exhibited adjusted average CEs of approximately 97.4 % and 96.9 %, respectively. However, considering the overall cycle life and interfacial integrity, ZnO@CF‐0.2 and ‐0.3 can be concluded to be significantly more stable for long‐term LMB operations. These findings confirm that while higher ZnO loading may benefit LIB performance, the facet‐dependent regulation is the decisive factor in ensuring the stability of lithium metal anodes

These results reveal that electrodes appearing stable during conventional LIB operation can become unstable when Li plating and stripping occur in the LMB mode, demonstrating that a change in the Li storage mechanism can fundamentally alter the relative stability hierarchy among the electrodes. To quantitatively link this behavior to structural features [29, 43, 44], we focused on the crystal planes whose exposure varied most significantly with ETW pulse duration. Among the detectable reflections, the (002) and (101) planes exhibited the most pronounced and systematic evolution; thus, we used their intensity ratio (*I_002_*/*I_101_*) as a descriptor of the relative exposure of the polar (002) and semipolar (101) facets. Figure 3e presents the (002)/(101) ratio for each ETW pulse duration, which can be correlated with the electrochemical behavior. Furthermore, the validity of this ratio as a representative of crystallographic texture was confirmed by calculating the texture coefficient (*T_c_*) in Table S3, which shows a consistent trend with the *I_002_*/*I_101_* evolution. When the half‐cell results were analyzed together with this ratio, a clear mode‐dependent trend emerged: electrodes with *I*_002_/*I*_101_ >1 (i.e., more (002) facets were exposed) were more stable in the LIB mode, whereas those with *I*_002_/*I*_101_< 1 (more (101) facets) maintained more stable cycling under LMB conditions.

This contrast underscores the intrinsic role of crystal orientation in dictating Li‐storage pathways. The polar (002) facet, although suitable for Li‐ion intercalation, did not sufficiently regulate metallic Li deposition, leading to interfacial instabilities of ZnO under LMB conditions. Conversely, the non‐polar (101) facet effectively provided a chemically compatible, electronically robust interface that moderated Li‐metal nucleation and growth, thereby stabilizing plating/stripping. Accordingly, the CE trend in the LIB mode correlated positively with the (002)/(101) ratio, whereas that in the LMB mode correlated negatively, strongly suggesting that the (101) facet provides a more stable interface for Li‐metal cycling.

Because the CF current collector may influence nucleation under the LMB‐type half‐cell window (from ∼1 V down to the Li‐plating potential), we further conducted pseudo‐symmetric‐cell tests (4 mAh cm^−2^ plated ZnO@CF vs. Li chip) to isolate the intrinsic Li reversibility (Figure 3f). Each ZnO@CF electrode was first pre‐loaded with 4 mAh cm^−2^ of Li and paired against a Li foil. Upon cycling at 1 mA cm^−2^ (1 mAh cm^−2^ per cycle), the pseudo‐symmetric cells reproduced the half‐cell trend (Figure 3c): (002)‐dominant ZnO@CF‐0.4 and ZnO@CF‐0.5 failed earlier and exhibited rising overpotentials, whereas (101)‐dominant ZnO@CF‐0.2 and ZnO@CF‐0.3 operated stably for >800 h. Notably, as the test approached 750 h, ZnO@CF‐0.2 (planar) showed slightly smaller overpotential fluctuations than ZnO@CF‐0.3 (3D particles), despite both being (101)‐dominant by XRD. This divergence is therefore attributed to morphology (planar vs. 3D) rather than facet identity. This finding indicates that achieving interfacial stability requires considering not only the crystallographic facet ((101) vs. (002)), which dictates the intrinsic interfacial reactivity, but also the physical morphology (planar vs. 3D particle), which is hypothesized to govern the electric field distribution and localized current densities, thereby influencing the overall plating/stripping kinetics. To further elucidate the origin of these performance variations, we performed comprehensive post‐cycling morphological analysis and symmetric cell rate capability tests (Figure S7). Consistently, the plating and stripping behavior observed in Figure S7 closely follows the trend established in Figure 3e, where ZnO@CF‐0.2 and ‐0.3 exhibit enhanced electrochemical stability compared to the other samples.

### 2.5 Mechanistic Analysis: Distinguishing the Effects of the Facets and Morphology

**FIGURE 4:**
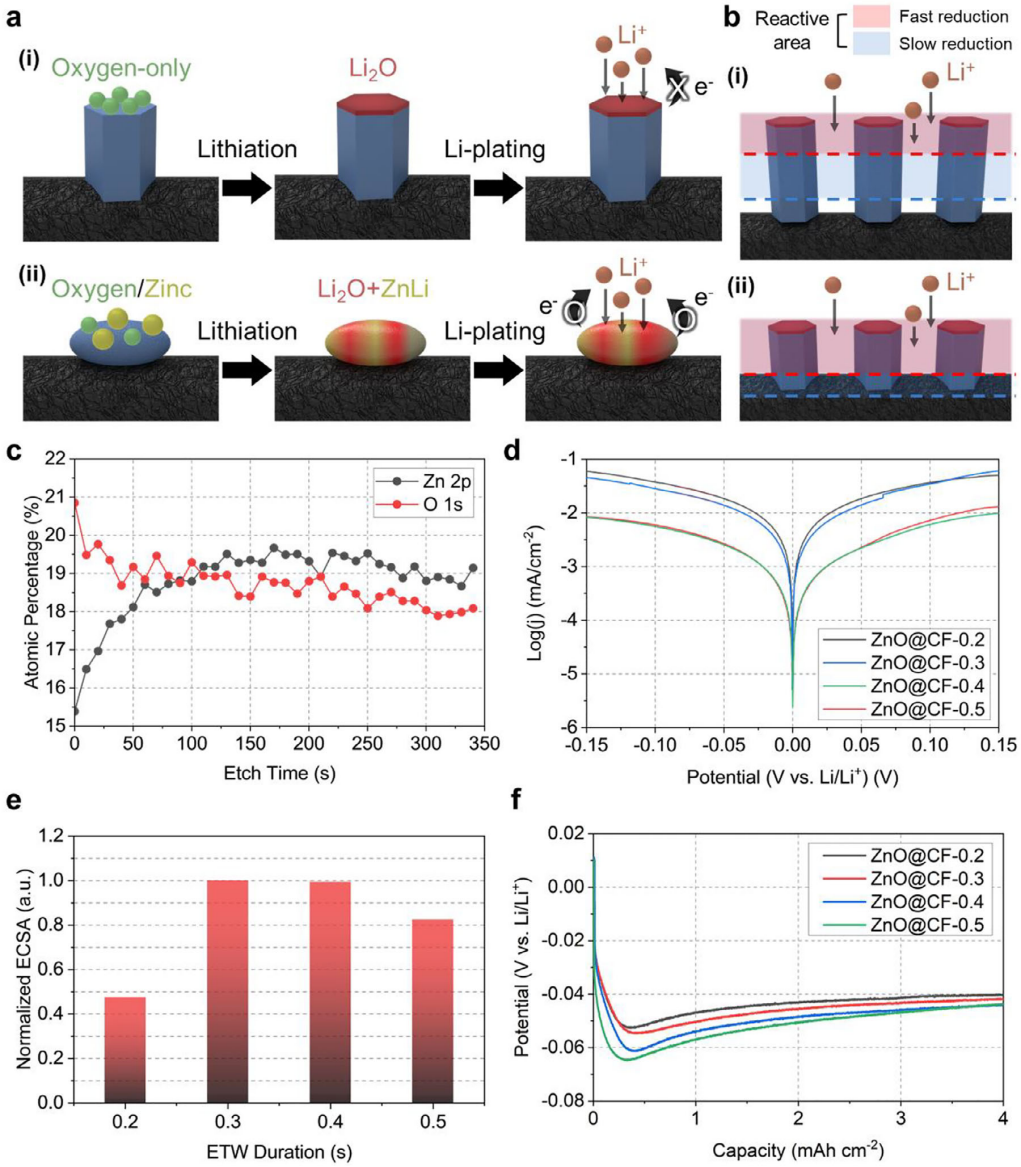
Proposed mechanisms governing Li behavior on ZnO@CF electrodes and corresponding experimental validation. (a) Schematic illustrations of two facet‐dependent lithiation and Li‐plating pathways: (i) oxygen‐terminated surfaces forming Li_2_O and (ii) mixed oxygen/zinc surfaces forming Li_2_O and ZnLi. (b) Morphology‐driven differences in the spatial distribution of reactive sites during Li deposition. (c) Atomic percentage variation obtained from XPS depth‐profile survey spectra of ZnO@CF‐0.4 collected during sequential Ar^+^ etching (35 cycles, 10 s per cycle; total 350 s; 2000 eV), showing the evolution of the Zn 2p and O 1s signals with depth. (d) Tafel plots of the ZnO@CF electrodes. (e) Normalized electrochemically active surface area (ECSA) extracted from the double‐layer capacitance method. (f) Voltage profiles during Li plating, showing overpotential.

To elucidate the mechanistic origins of the observed electrochemical stability, we disentangled the independent effects of crystal facet and physical morphology on Li plating/stripping kinetics. Two complementary hypotheses were proposed, related to facet configuration and morphology (Figure 4a,b). First, we hypothesize that the crystal facet dictates the electronic and ionic characteristics of the ZnO–Li interface (Figure 4a). In wurtzite ZnO, the polar (002) facet, terminated exclusively by either Zn or O atoms, exhibits asymmetric charge distribution and strong dipole moments. Under O‐rich (ambient‐air) ETW conditions, the O‐terminated (002) surface becomes thermodynamically favored [45, 46, 47, 48]. This oxygen‐rich surface could easily form an electronically insulating Li_2_O layer during lithiation [2]. The (002) facet is composed exclusively of either Zn or O atoms, and polar O‐ or Zn‐terminated facets more effectively impede interfacial charge transfer than non‐polar facets. In this study, ETW processing was carried out under the oxygen‐rich ambient atmosphere, which facilitated the formation of O‐terminated (002) facets. These facets, in turn, acted as a significant barrier to electron exchange after the conversion reaction, particularly during the onset of Li plating.

Second, we hypothesized a morphology‐dependent kinetic area. Li plating proceeds significantly faster than intercalation or conversion reactions, making concentration polarization more likely under practical operation conditions [49, 50, 51]. Under such fast kinetics, a 2D‐like planar morphology provides more efficient utilization of the reaction area than a complex 3D architecture [52], as schematically illustrated in Figure 4b. If the 3D architecture did not affect the electrochemical behavior, ZnO@CF‐0.2 and ZnO@CF‐0.3, despite having similar facet fractions, would exhibit comparable intrinsic exchange current densities (j_0,intrinsic_). Any deviation between them would therefore be attributable to morphological differences.

Figure 4c and Figure S8 present the depth‐dependent atomic percentage of oxygen within the ZnO@CF‐0.4 electrode via XPS depth‐profile survey spectra, confirming that a higher oxygen concentration existed near the outer surface, corroborating the formation of O‐terminated ZnO facets. Conversely, after lithiation, metallic Zn remained on the non‐polar (101) facet, featuring alternating Zn and O atoms [39, 42]. This conductive and lithiophilic Zn (capable of forming Li–Zn alloys) provided a superior pathway for electron exchange, thus facilitating stable Li nucleation [9, 20, 21]. Furthermore, the XPS analysis conducted just prior to the onset of Li plating (Figure S9) provides strong evidence that samples with higher (002) facet intensities indeed exhibit a greater accumulation of Li_2_O on their surfaces, further supporting our hypothesis regarding facet‐dependent interfacial evolution.

These two hypotheses were validated by detailed electrochemical kinetic analyses. Tafel plots were constructed to compare the activities and reaction mechanisms during Li plating and stripping (Figure 4d; Figure S10 and Table S4). The linear regions of the plots exhibited different Tafel slopes (*β*), with the (101)‐dominant samples showing values near 210 mV dec^−1^, whereas the (002)‐dominant samples exhibited steeper slopes of around 250 mV dec^−1^ during plating. This indicates facet‐dependent variations in the charge‐transfer coefficient (*α*), and therefore in the activation barrier for Li nucleation, supporting the facet hypothesis. Furthermore, the exchange current densities (j_0_) of the (101)‐dominant electrodes were approximately 6.45 times higher than those of the (002)‐dominant ones, consistent with their more conductive and lithiophilic interfacial chemistry.

Electrochemical impedance spectroscopy (EIS) measurements further supported these results (Figures S11 and S12). Figure S12a shows the evolution of the average charge‐transfer resistance (R_ct_), estimated from the semicircle diameter, in both the LIB operating region and at 0 V vs. Li/Li^+^ over four consecutive EIS measurements. Once the potential reached 0 V vs. Li/Li^+^, at which point intercalation and conversion are largely completed, the (002)‐dominant samples exhibited a noticeable increase in R_ct_. This behavior is consistent with the formation of a more resistive interfacial layer on the polar (002) surface, in line with our first hypothesis. Figure S12b presents the evolution of R_ct_ from EIS spectra measured under different bias potentials during Li plating. As plating initiated, the reaction proceeded more rapidly than intercalation or conversion, leading to a characteristic decrease in R_ct_. The bias potential at which this decrease began varied among the samples, indicating that the onset of Li plating was facet dependent. The (101)‐dominant electrodes exhibited this decrease at lower bias, whereas the (002)‐dominant electrodes required a higher driving force to reach the same transition. Furthermore, a comparison of the EIS profiles at 0 V vs. Li/Li^+^ in the first cycle with the in situ galvanostatic EIS (GEIS) results obtained during plating at the fifth cycle (1 mA cm^−2^) further confirms that electrodes with minimal (002) exposure maintain a more stable interface during practical plating operations (Figure S12c,d).

Furthermore, to determine *j_0,intrinsic_*, we evaluated the electrochemically active surface area (ECSA) by extracting the double‐layer capacitance (C_dl_) from ECSA cyclic voltammetry (CV) measurements. Assuming identical intrinsic activity per unit ZnO surface, the resulting ECSA values were used to normalize the exchange current densities (Figure 4e; Figure S13). Considering the SEM images and BET (Figure 3e), although the overall trend in area did not change significantly, the relative values varied substantially. This suggests that the differences were likely attributable to morphological effects, because non‐Faradaic processes respond more sensitively to changes in geometrically accessible surface area. Based on the relative ECSA values, the relative *j_0,intrinsic_* values were determined to be 13.19, 5.26, 3.56, and 3.84 for ZnO@CF‐0.2, ‐0.3, ‐0.4, and ‐0.5, respectively. ZnO@CF‐0.4 and ‐0.5, which share similar morphology and facet dominance, exhibited only a small difference, whereas ZnO@CF‐0.2 and ‐0.3 showed a non‐negligible ∼2.5 fold difference. This result supports our second hypothesis that the 3D architecture can exert a non‐negligible influence on the lithium plating reaction.

Consequently, both the facet orientation and morphology introduced measurable variations in interfacial charge‐transfer resistance during Li plating. During galvanostatic Li plating, the nucleation potential increased as the dominance of the (002) facet became larger (Figure 4f). Accordingly, the (002)‐dominant samples (ZnO@CF‐0.4 and ZnO@CF‐0.5) exhibited much higher overpotentials, reflecting their poor plating reactivity (low *j*_0_). Among the (101)‐dominant samples, ZnO@CF‐0.2 showed a lower overpotential than ZnO@CF‐0.3, consistent with its higher *j*_0_ and more favorable morphology. Nonetheless, all ZnO‐containing electrodes outperformed the bare CF substrate, confirming the intrinsic lithiophilicity of ZnO.

According to the above discussion, this behavior is likely attributed to coverage of the (002) facet by Li_2_O, which increases the apparent activation energy (Δ*G*_app_) for nucleation. Because reactions can occur on all exposed facets, Δ*G*_app_can be expressed as:

(1) $$\Delta G_{\mathrm{app}}∼\frac{1}{\eta^{2}},$$

(2) $$\Delta \;G_{app}=\theta_{002}\cdot \Delta G_{\left(002\right)}+\left(1-\theta_{002}\right)\cdot \Delta G_{non-002,app}$$

where η is the overpotential, θ_002_ denotes the area fraction of the (002) facet, Δ*G*_(002)_ is the activation energy associated with the (002) facet, and Δ*G*_non‐002_ represents the apparent activation energy of all non‐(002) facets collectively. Because 𝜃_002_ is proportional to the normalized XRD (002) diffraction peak intensity 𝐼_002_ / Σ𝐼_hkl_, if the (002) facet strongly governs nucleation as proposed, the following linear relation is expected:

(3) $$I_{002}∼\frac{1}{\eta^{2}}$$

Figure S14 shows the linear fitting of *I*_002_ as a function of 1/η^2^, confirming their linearity. The coefficient of determination (R^2^ = 0.96) indicates that the two variables are well described by the proposed relationship.

This analysis decoupled and identified two key factors governing the cycling stability of Li metal. The (101) crystal facet imparted intrinsically superior electronic conductivity compared with the (002) facet, while the 2D planar morphology enabled greater kinetic accessibility by providing a more effectively utilized electrochemically active surface area (and consequently a higher j_0_) under LMB‐type operation. The pronounced difference in intrinsic reactivity between the (101)‐ and (002)‐dominant electrodes demonstrates that facet‐dependent mechanisms play a primary role in determining the baseline interfacial behavior. Considering ZnO@CF‐0.3 and ZnO@CF‐0.4, which exhibited comparable geometric surface area and ECSA, their markedly different intrinsic exchange current densities further indicate that facet orientation is the primary determinant of intrinsic reactivity, whereas morphology acts as a secondary factor that modulates how effectively this intrinsic reactivity is expressed during practical cycling.

### 2.6 Evaluation of ZnO@CF Electrodes as LMB Anodes

**FIGURE 5:**
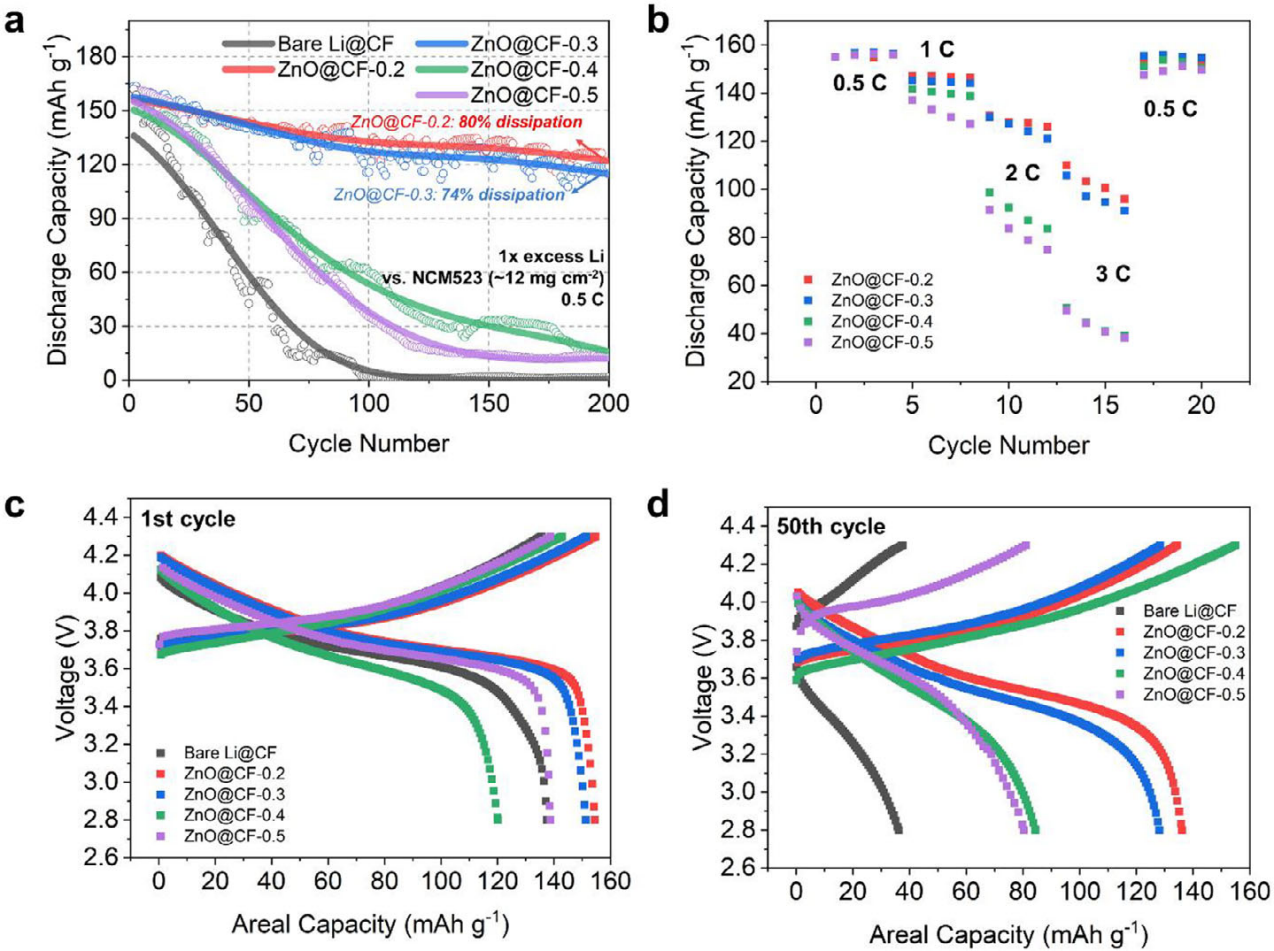
Electrochemical performance of ZnO@CF anodes paired with NCM523 cathodes in a full‐cell configuration. (a) Long‐term cycling stability at 0.5 C, with specific capacity calculated based on the active mass of the NCM523 cathode (Bare Li@CF denotes the control sample without ZnO nanostructures). (b) Rate capability performance of the full cells cycled at various C‐rates ranging from 0.5 C to 3 C. (c,d) Representative charge–discharge voltage profiles at the (c) first cycle and (d) 50th cycle, illustrating the evolution of voltage hysteresis and capacity retention. All full cells were assembled with a pre‐deposited Li reservoir of 4 mAh cm^−2^.

Building upon the mechanistic understanding that the (101) facet provides intrinsically conductive and lithiophilic interfacial sites while the 2D planar morphology facilitates uniform current distribution, the electrochemical performance of the engineered ZnO@CF electrodes was evaluated under practical full‐cell configurations. In this design, ZnO@CF functions as a chemically and structurally stable interfacial regulator rather than as an active host material, aiming to stabilize Li‐metal deposition and mitigate interfacial degradation during long‐term operation. Full cells were assembled using a commercial NCM523 cathode with an areal capacity of approximately 1.8 mAh cm^−2^ (Figure 5). To ensure a consistent Li inventory and prevent premature depletion of metallic Li, a Li reservoir of 4 mAh cm^−2^ (corresponding to ∼100 % excess relative to the cathode capacity) was pre‐deposited onto each ZnO@CF electrode prior to assembly, and the cells were cycled at 0.5 C.

The full‐cell results clearly validated the mechanistic framework established from the half‐ and symmetric‐cell studies. All ZnO‐coated electrodes exhibited significantly improved cycling stability compared with the bare CF substrate, confirming that ZnO effectively acts as an interfacial regulator capable of stabilizing Li‐metal plating and mitigating side reactions. Nevertheless, distinct differences appeared among the ZnO@CF samples depending on their crystal orientation and morphology. The (002)‐dominant electrodes (ZnO@CF‐0.4 and ZnO@CF‐0.5) displayed rapid capacity fading and progressively increased polarization during cycling. This degradation is clearly visualized in the evolution of the charge–discharge voltage profiles (Figure 5c,d); unlike the stable hysteresis observed in ZnO@CF‐0.2, the control samples showed a substantial increase in overpotential from the first to the 50th cycle. This behavior is consistent with the formation of an electronically insulating Li_2_O‐rich surface layer on O‐terminated (002) facets, which increases interfacial impedance and induces uneven Li nucleation.

In contrast, the (101)‐dominant electrodes (ZnO@CF‐0.2 and ZnO@CF‐0.3) demonstrated much higher reversibility and more stable voltage profiles. Their Coulombic efficiencies remained high throughout extended cycling, with only minor polarization growth, indicating uniform Li plating and stripping. These results corroborate that the (101) facets provide conductive and lithiophilic nucleation sites while the planar surface morphology ensures homogeneous current distribution and suppresses dendritic growth.

Among the four electrodes, ZnO@CF‐0.2 exhibited the most stable cycling performance, attributed to the synergistic combination of its (101)‐dominated crystal orientation and smooth 2D surface morphology. This configuration minimized charge‐transfer resistance and overpotential, maintaining stable voltage hysteresis and high reversibility even under practical cycling conditions. Furthermore, the facet‐engineered advantage was confirmed by rate capability tests (Figure 5b), where the ZnO@CF‐0.2 anode sustained high specific capacities across a wide range of current densities (0.5 C to 3 C), significantly outperforming the sluggish kinetics of the (002)‐dominant and bare CF electrodes. The slightly rougher 3D surface of ZnO@CF‐0.3 resulted in somewhat larger polarization, consistent with its lower exchange current described in Section 2.5. Nevertheless, both (101)‐dominant samples significantly outperformed the (002)‐dominant ones and the pristine CF, confirming that interfacial stabilization in Li‐metal anodes depends critically on both crystallographic and morphological control.

Overall, these findings demonstrate that intrinsic interfacial reactivity, dictated by the exposed crystal facets, and kinetic accessibility, governed by surface morphology, act together to determine long‐term electrochemical stability. The conductive and lithiophilic (101) facet enables facile charge transfer and uniform Li nucleation, while the planar configuration minimizes local current hotspots and mechanical stress accumulation. Consequently, the optimized ZnO@CF‐0.2 electrode functions as a self‐regulating Li‐metal interface capable of stable and reversible cycling without the need for artificial SEI layers or external additives. This integrated facet–morphology design concept provides a generalizable route toward practical and durable next‐generation Li‐metal batteries.

## 3 Conclusion

In summary, this study redefined ZnO not as a conventional active material but as a facet‐engineered interfacial regulator that can stabilize Li‐metal anodes through atomic‐scale and morphological control. Using electrothermal‐wave (ETW) processing, we precisely modulated the diffusion kinetics of Zn and O species, enabling selective tuning of both the exposed crystal facets and the nanostructural morphology. This controllable platform allowed us to decouple the effects of crystallographic orientation and physical geometry on interfacial stability, revealing that, in contrast to its behavior in conventional LIBs, the (101) facet of ZnO provides superior interfacial stability in LMB systems compared with the polar (002) facet. Mechanistic analyses combining Tafel, EIS, and overpotential measurements clarified the origin of this difference. The (101) facet offered intrinsically higher electronic conductivity and lithiophilicity by maintaining a metallic Zn interface favorable for uniform Li nucleation, whereas the O‐terminated (002) facet readily formed an electronically insulating Li_2_O layer that hindered charge transfer and promoted nonuniform deposition. Simultaneously, the 2D planar morphology provided a kinetically advantageous ECSA and higher exchange current density (j_0_) for rapid Li plating compared with the 3D particle morphology. The optimized ZnO@CF‐0.2 electrode, which uniquely combined the (101)‐dominant facet with a planar architecture, exhibited the most stable performance in both half‐cell and full‐cell configurations. When paired with a commercial NCM523 cathode, it maintained stable voltage profiles and excellent reversibility over extended cycling, validating the synergistic role of crystallographic and morphological control in achieving interfacial stability. Overall, this work establishes a mechanistic framework in which facet‐dependent interfacial reactivity and morphology‐dependent kinetic regulation act cooperatively to stabilize Li‐metal electrodes. Beyond revising the conventional view of ZnO as a low‐performance anode, our findings demonstrate that oxides can serve as chemically active interfacial regulators when their atomic structure and geometry are rationally tailored. This concept provides a general and broadly applicable strategy for designing durable, high‐efficiency interfaces in Li‐metal and other reactive‐metal battery systems.

## 4 Materials and Methods

### 4.1 Chemicals

Zinc nitrate hexahydrate, 1.0 m LiPF_6_ in ethylene carbonate (EC) and diethyl carbonate (DEC) (1:1, v/v), and lithium bis(trifluoromethanesulfonyl)imide (LiTFSI) salt were purchased from Sigma–Aldrich. NCM523 was purchased from MTI Korea. CF sheets (HCP030, thickness 300 µm) were purchased from WizMac (Republic of Korea).

### 4.2 Preparation of ZnO@CF Electrodes

The CF sheets were cut into rectangles with dimensions of 2 × 1 cm^2^. Zinc nitrate (Zn(NO_3_)_2_·6H_2_O) was dissolved in a 75 % ethanol solution to prepare a 0.1 m precursor solution. Approximately 50 µL of this solution was drop‐cast onto each CF sheet, and the coated substrates were dried in ambient air for 24 h to ensure the solvent completely evaporated. The dried samples were subjected to ETW treatment using a DC power supply (ITECH, IT6512D). An electric current of 25 A at 20 V was applied to the specimens for various durations of 0.1, 0.2, 0.3, 0.4, and 0.5 s to induce thermal decomposition and the subsequent formation of ZnO nanostructures. The consistency of ZnO loading was verified by comparing the measured mass gain with the theoretical yield. Control experiments using bare CF substrates under identical ETW conditions were performed to calibrate the substrate's weight changes. The resulting ZnO loading was confirmed to be uniform across all samples, as the peak ETW temperatures (∼1600 K) remained well below the melting point of ZnO (∼2250 K), preventing any significant mass loss.

### 4.3 Characterization of ZnO@CF Electrodes

The surface morphologies of the ZnO@CF electrodes were investigated using field‐emission SEM (FEI, Model Quanta 250 FEG; JEOL, Model JSM‐6701F). The chemical and elemental compositions were examined using XRD (Rigaku, SmartLab) and EDX (FEI, Tecnai G2 F30ST), respectively. Nitrogen adsorption measurements were performed to evaluate the specific surface area using the BET method, after degassing the samples at 250°C for 4 h under high vacuum. The measurements were conducted on film‐shaped samples (diameter: 4 mm, total mass: ∼0.4 g each) to preserve the as‐fabricated electrode configuration.

### 4.4 Electrochemical Characterization of ZnO@CF Electrodes in Half‐Cells

The electrochemical measurements were performed on CR2032 coin cells. First, the fabricated film was cut into a circle with a diameter of 11 mm for use as an electrode. For the half‐cell plating and stripping tests, the prepared circular electrode and a 10 nm‐thick Celgard polyethylene separator, a Li‐metal disc (diameter of 11 mm), and 1 m LiTFSI in 1,3‐dioxolane and 1,2‐dimethoxyethane (1:1, v/v) with 2 wt. % LiNO_3_ additive was used as the working electrode, separator, reference/counter electrode, and electrolyte, respectively. Li was plated onto the circular film electrode for 4 h at 1 mA cm^−2^ to examine the symmetric cell test. A symmetric cell was fabricated using two Li‐plated electrodes and the same materials as those employed in the half‐cell configuration.

### 4.5 EIS Test

EIS measurements were carried out using a potentiostat (ZIVE SP1, WonATech) in a half‐cell configuration. To investigate the potential‐dependent interfacial evolution, impedance spectra were recorded at potentials of 0.5, 0.1, and 0 V (vs. Li/Li^+^) over a frequency range from 10^5^ to 0.1 Hz. Furthermore, to evaluate the charge transfer kinetics under dynamic Li‐plating conditions, bias‐dependent PEIS measurements were conducted at 0 V (vs. Li/Li^+^) with applied cathodic biases of 20, 50, and 80 mV. These bias‐controlled measurements were performed in a frequency range from 10^5^ to 1 Hz. The obtained Nyquist plots were fitted to an equivalent circuit model to extract the charge transfer resistance (*R*_ct_).

### 4.6 CVs with ZnO@CF Electrodes

CV measurements were conducted in a half‐cell configuration to obtain both the Tafel plots and the electrochemically active surface area (ECSA). For the Tafel analysis, the open‐circuit voltage (OCV) was first adjusted to 0 V, and the cell was pre‐cycled at an areal capacity of 0.5 mAh cm^−2^ to ensure electrochemical stabilization. Subsequently, the potential was swept between −0.175 V and 0.175 V at a scan rate of 0.2 mV s^−1^. ECSA was evaluated in the same half‐cell configuration by performing CV measurements in a potential window near the OCV where Faradaic contributions are negligible (2.10–2.17 V vs. Li/Li^+^). The scan rate was varied from 1, 2, 3, 4, to 5 mV s^−1^, and the capacitive current density (Δ*j* = (*j*_anodic_ − *j*_cathodic_)/2 at a fixed potential) was plotted as a function of scan rate. The slope of this linear relationship was used to determine the double‐layer capacitance (*C*_dl_) according to *j* = *v C*_dl_, following the double‐layer capacitance (*C*_dl_) method, from which the ECSA was estimated.

### 4.7 Electrochemical Full Cells with ZnO@CF Electrodes

Full cells were fabricated using a Li‐plated electrode, NCM523, and 1 m LiPF_6_ in EC/DEC (v/v 1:1) with 5 % fluoroethylene carbonate additive as the anode, cathode, and electrolyte, respectively. Prior to full‐cell assembly, lithium was electrodeposited onto the ZnO@CF electrode at a current density of 1 mA cm^−2^ to a total areal capacity of 4 mAh cm^−2^ (corresponding to an N/P ratio of ∼2.2). Each full cell was assembled with 100 µL of electrolyte to ensure sufficient ionic conductivity. All cells were fabricated in an Ar‐filled glove box (H_2_O< 0.1 ppm and O_2_< 0.1 ppm), and a WBCS3000 battery tester (WonATech) and a ZIVE SP1 (WonATech) were used for all electrochemical analyses.

## Conflicts of Interest

The authors declare no conflict of interest.

## Supporting information




**Supporting File**: smll73001‐sup‐0001‐SuppMat.docx.

## Data Availability

The data that support the findings of this study are available from the corresponding author upon reasonable request.
