# Supplementary material for: Facet‐Engineered ZnO as an Interfacial Regulator for Stable Lithium Metal Anodes
Source: Small. 2026 Mar 1;22(28):e14924. doi: 10.1002/smll.202514924 (PMC13181513; doi:10.1002/smll.202514924)
Supplement: Supplementary file 1 — Supporting File: smll73001‐sup‐0001‐SuppMat.docx. [file SMLL-22-e14924-s001.docx]

Supporting Information

Facet-Engineered ZnO as an Interfacial Regulator for Stable Lithium Metal Anodes

Kyungmin Kim, Seonghyun Park, Hwanju Lim, Jeongwoo Lee, Sohyung Jiong, Xinqi Chen, Dahyun Daniel Lim, Byungseok Seo*, and Wonjoon Choi*

K. Kim, S. Park, H. Lim, J. Lee, S. Jiong, D. Lim, B. Seo, W. Choi
School of Mechanical Engineering, Korea University
Seoul, 02841, Republic of Korea
E-mail: wojchoi@korea.ac.kr

X. Chen
Department of Mechanical Engineering, Northwestern University
Evanston, IL 60208, United States

B. Seo, X. Chen
The NUANCE Center, Northwestern University
Evanston, IL 60208, United States

E-mail: byungseok.seo@northwestern.edu

Kyungmin Kim and Seonghyun Park contributed equally to this work.


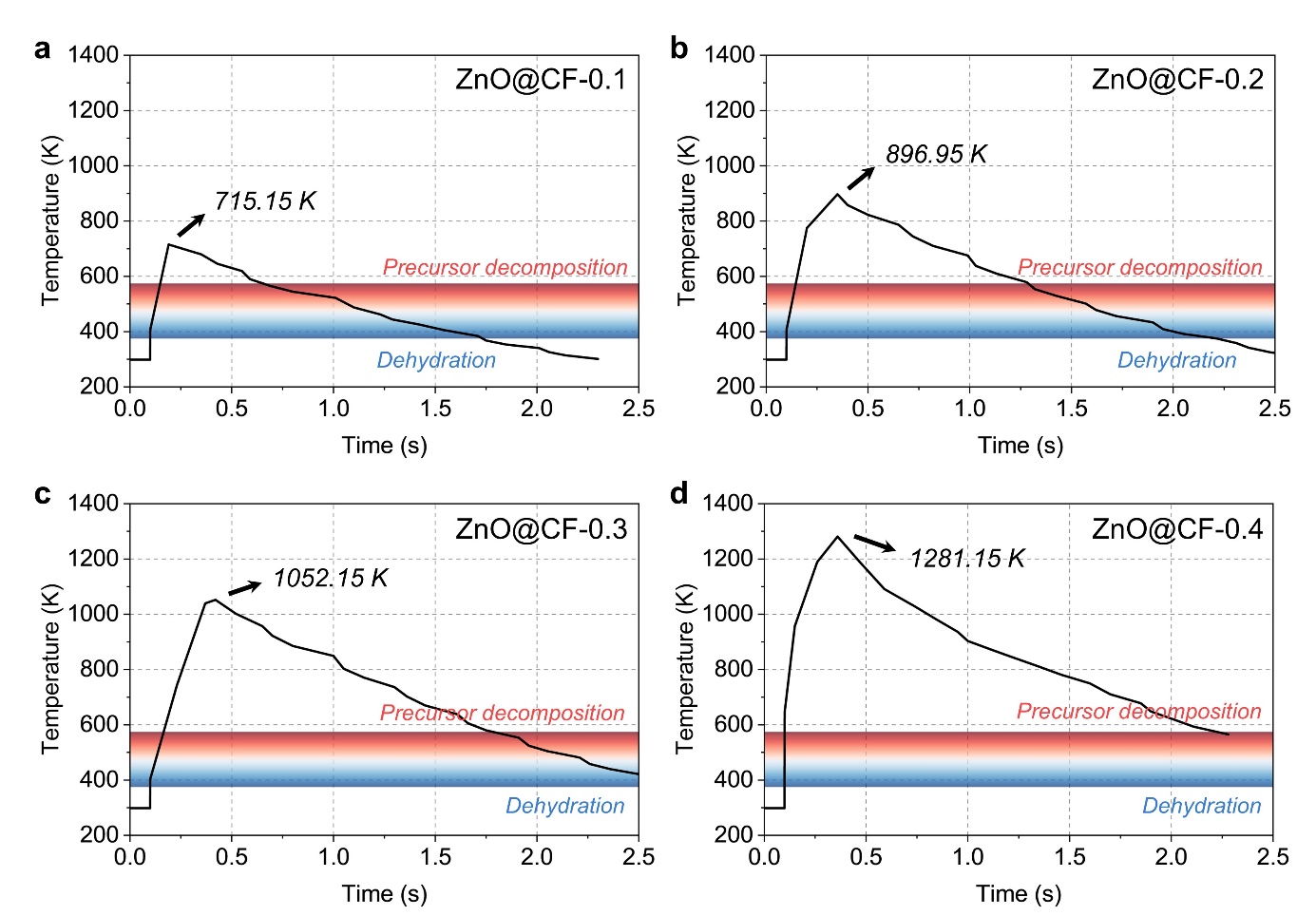


**Figure S1.** Real-time temperature profiles of (a) ZnO@CF-0.1, (b) ZnO@CF-0.2, (c) ZnO@CF-0.3, and (d) ZnO@CF-0.4 during ETW processing. Each curve represents a single ETW pulse (500 W) with varying durations, showing the corresponding peak temperatures and the temperature ranges associated with precursor dehydration and decomposition.

**Table S1.** Full width at half maximum (FWHM) values of the XRD reflections corresponding to the (100), (002), and (101) planes for the ZnO@CF samples processed under different ETW pulse durations.

| **(hkl)** | **ZnO@CF-0.1** | **ZnO@CF-0.2** | **ZnO@CF-0.3** | **ZnO@CF-0.4** | **ZnO@CF-0.5** |
| --- | --- | --- | --- | --- | --- |
| **(100)** | 0.488 | 0.405 | 0.392 | 0.341 | 0.328 |
| **(002)** | 0.499 | 0.425 | 0.404 | 0.335 | 0.329 |
| **(101)** | 0.549 | 0.453 | 0.427 | 0.361 | 0.332 |

**Table S2.** Nitrogen atomic percentages of the ZnO@CF samples measured by XPS.

|  | **ZnO@CF-0.1** | **ZnO@CF-0.2** | **ZnO@CF-0.3** | **ZnO@CF-0.4** | **ZnO@CF-0.5** |
| --- | --- | --- | --- | --- | --- |
| **N atomic percentage (%)** | 4.54 | 1.90 | 2.28 | 0.82 | 0.98 |


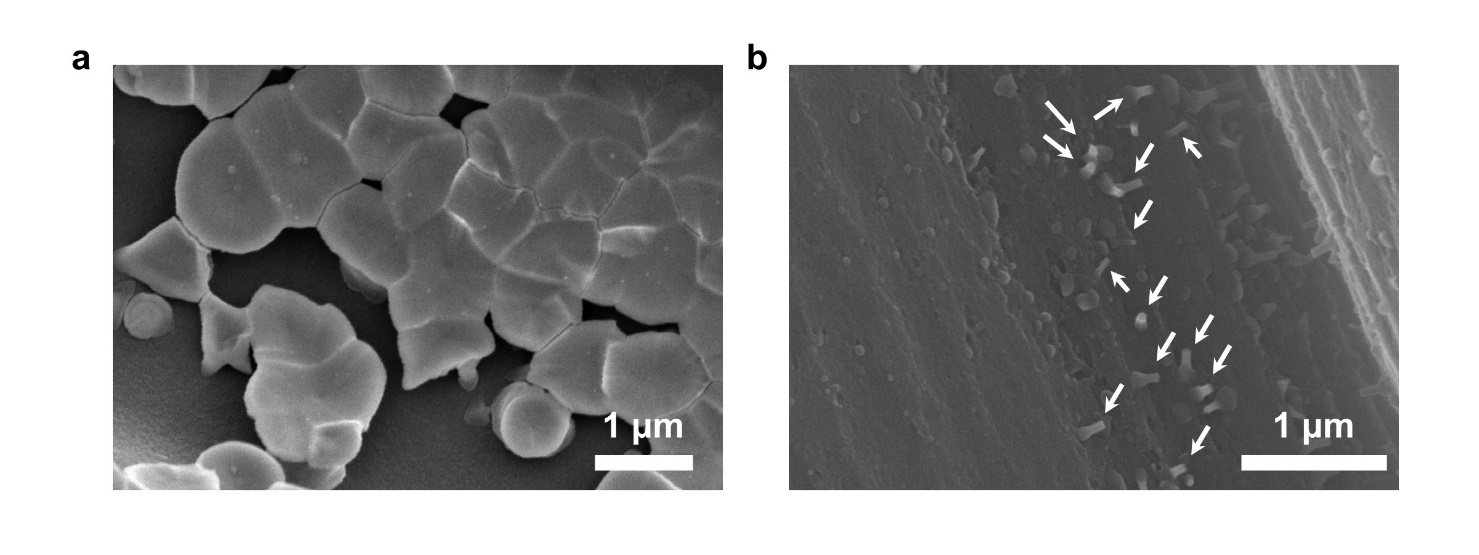


**Figure S2.** Scanning electron microscope (SEM) images confirming the threshold condition for hexagonal ZnO crystallite formation. (a) Four repeated ETW pulses of 0.2 s and (b) a 0.2 s pulse followed by a 0.3 s pulse (exceeding the threshold), with the emergence of hexagonal grains highlighted by arrows.


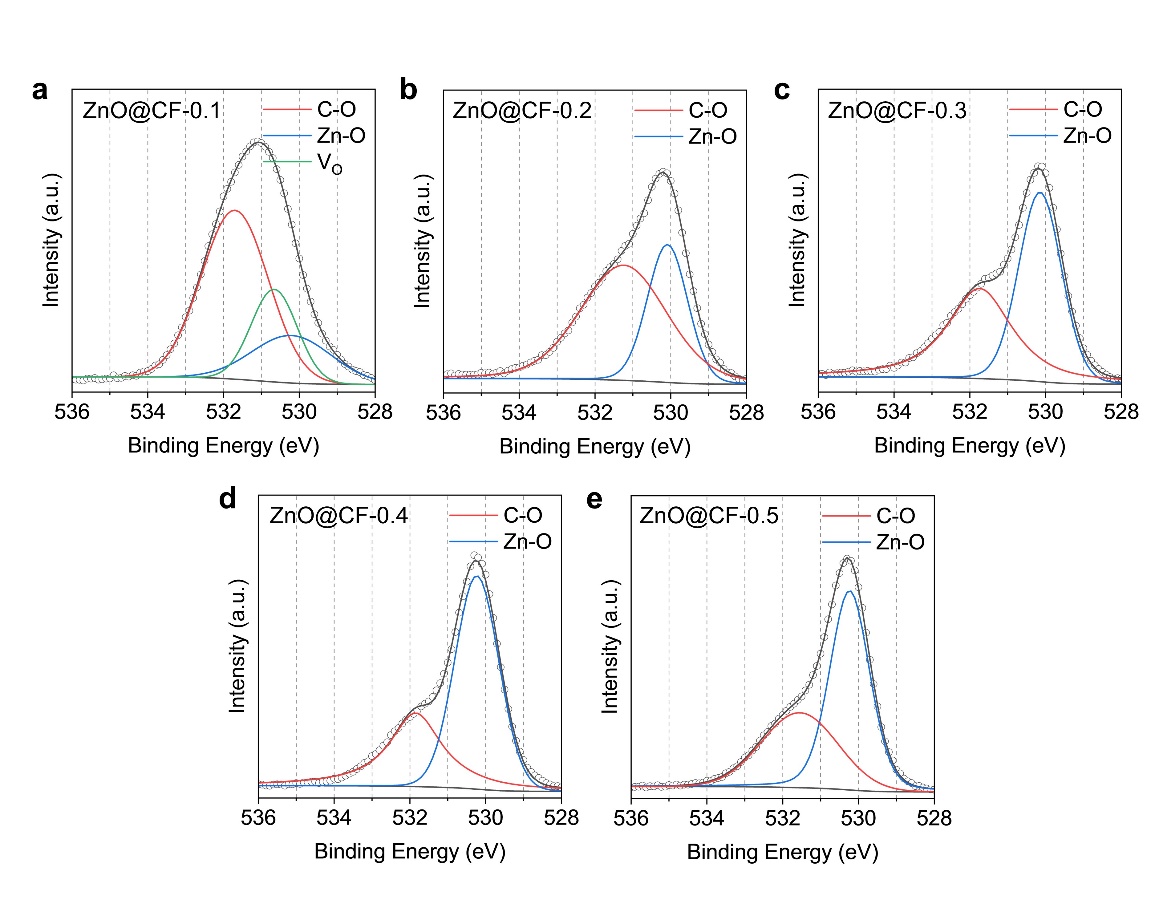


**Figure S3.** X-ray photoelectron spectroscopy (XPS) O 1s spectra of the ZnO@CF composites. (a) ZnO@CF-0.1, (b) ZnO@CF-0.2, (c) ZnO@CF-0.3, (d) ZnO@CF-0.4, and (e) ZnO@CF-0.5, with deconvoluted peaks corresponding to C–O, Zn–O, and oxygen-vacancy–related components.


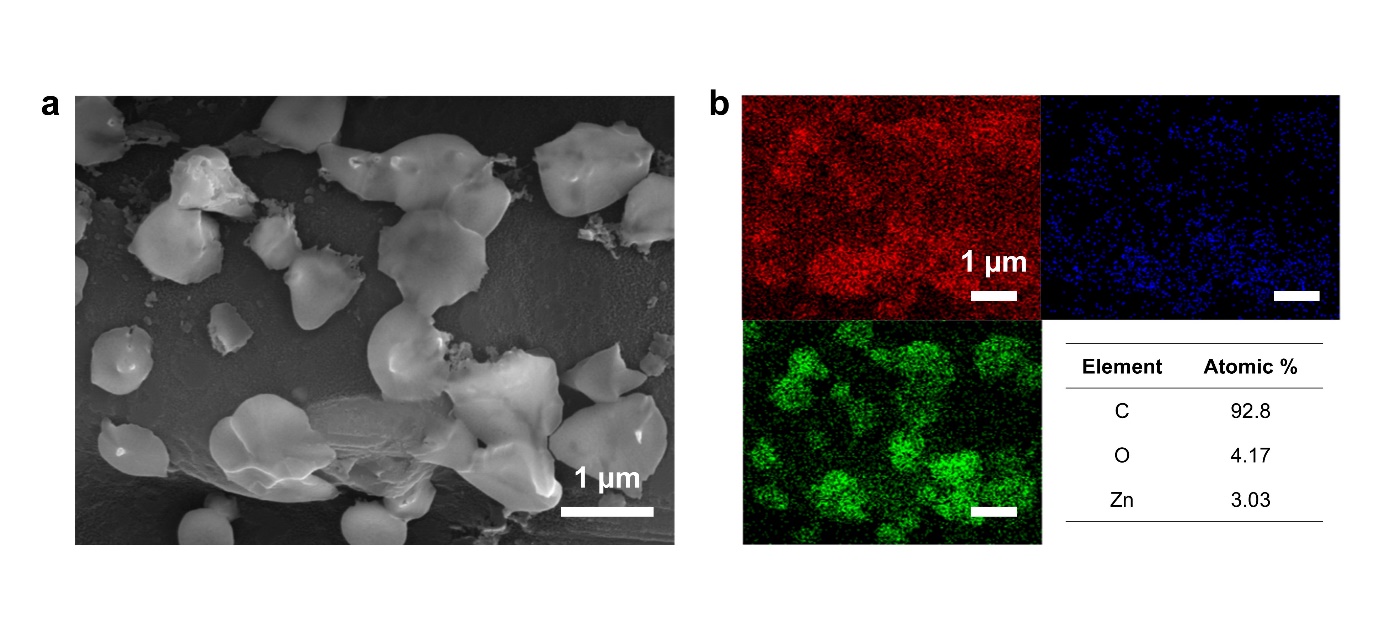


**Figure S4.** Energy-dispersive X-ray spectroscopy (EDX) analysis of ZnO@CF-0.2. (a) SEM image of the surface and (b) elemental mapping of C, O, and Zn with corresponding atomic percentages.


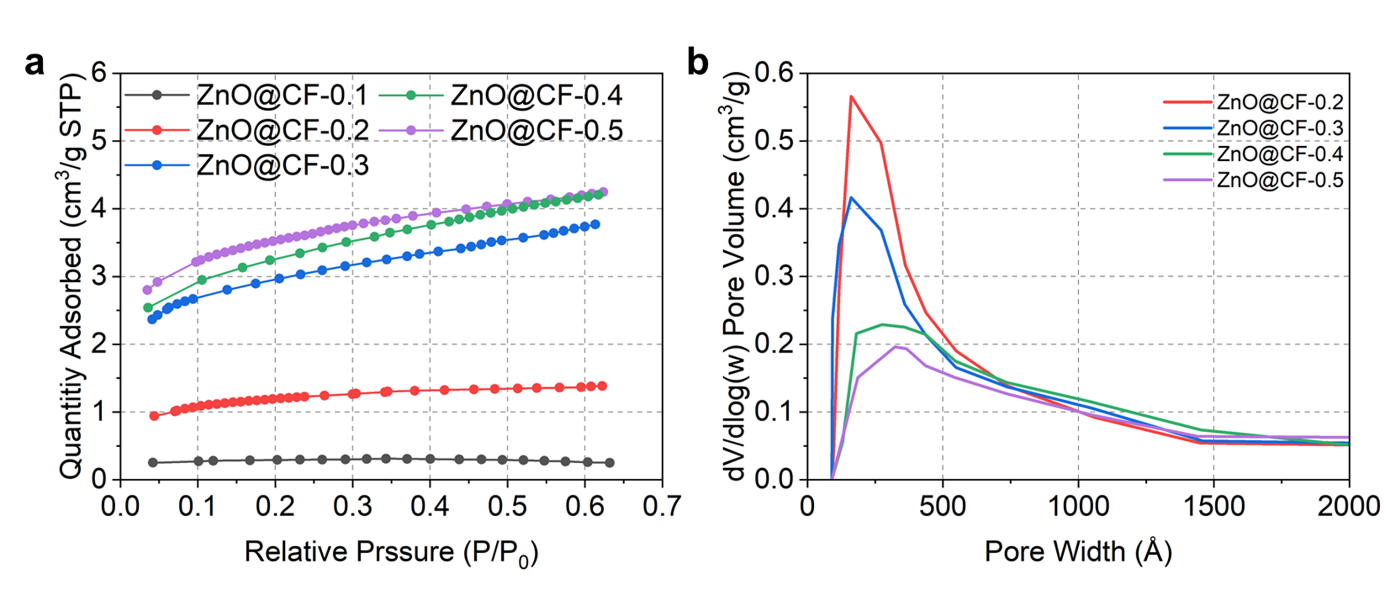


**Figure S5.** Nitrogen adsorption analysis for specific surface area and pore structure. (a) N_2_ adsorption isotherms of ZnO@CF-0.1 to -0.5, and (b) Barrett-Joyner-Halenda (BJH) pore size distribution of ZnO@CF-0.2, to -0.5. The pore size distribution of ZnO@CF-0.1 is not shown in (b) due to the high signal-to-noise ratio arising from its low specific surface area. All measurements were conducted on film-shaped samples (diameter: 4 mm, total mass: ~0.4 g each) after a degassing process at 250 °C for 4 h under high vacuum.


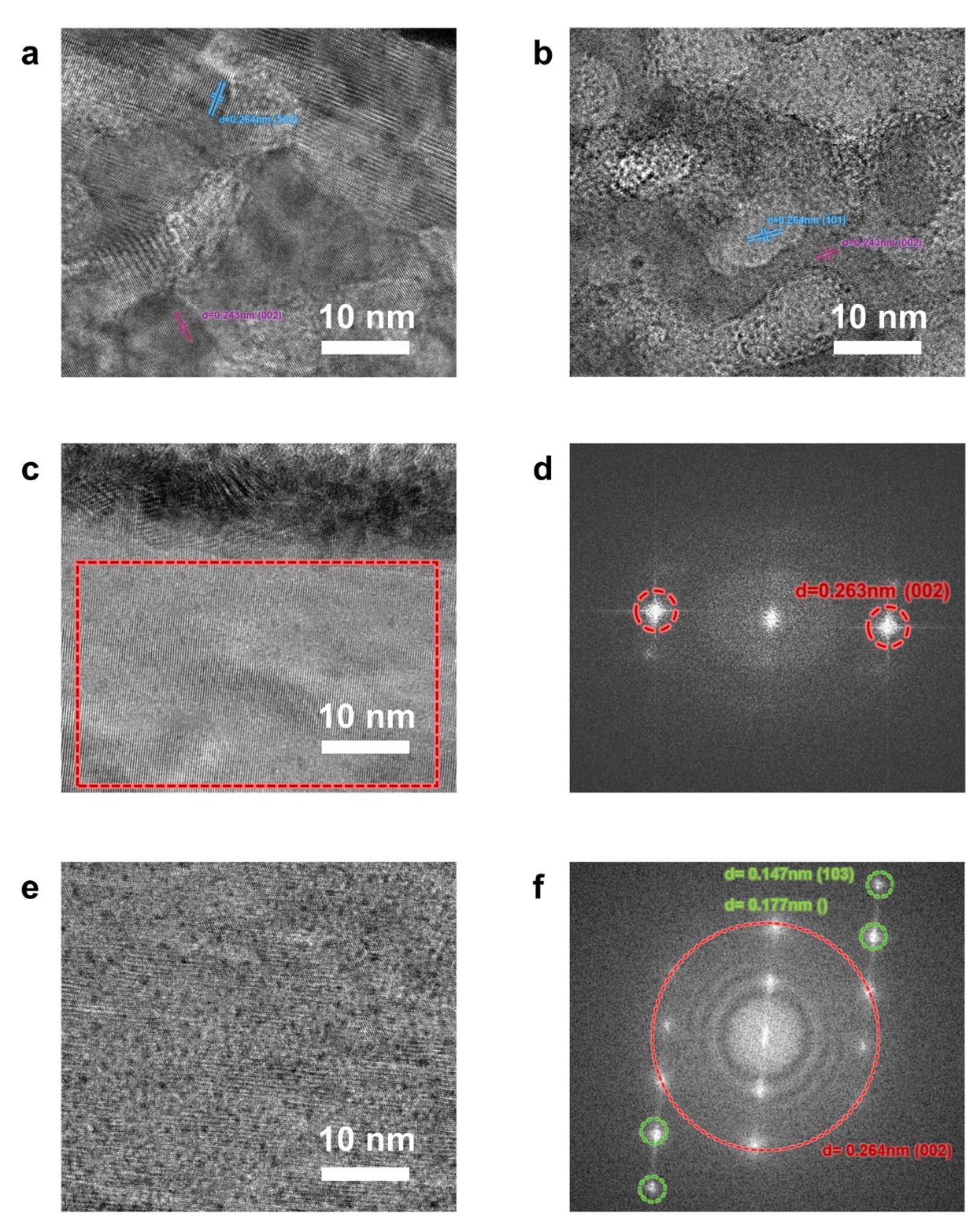


**Figure S6.** Nanoscale structural evolution and crystallographic orientation of ZnO nanostructures. (a, b) High-resolution transmission electron microscopy (HR-TEM) images of (a) ZnO@CF-0.2 and (b) ZnO@CF-0.3, revealing the coexistence of randomly distributed lattice fringes corresponding to both the (101) and (002) planes. Structural analysis of ZnO@CF-0.4, displaying (c) an HR-TEM image with a large, highly ordered single-crystal-like region (marked by the red dashed box) and (d) the corresponding selected area electron diffraction (SAED) pattern, confirming the dominance of the (002) crystallographic orientation. Analysis of ZnO@CF-0.5, showing (e) the uniform HR-TEM lattice structure and (f) the corresponding SAED pattern, where the high intensity and predominance of (002)-related diffraction spots indicate a strong preferred orientation along the c-axis.

**Table S3.** Detailed XRD peak intensity ratios and texture coefficients (*T_c_*)calculation.

|  | Plane  (hkl) | Normalized Intensity  (*I*) | Standard Intensity  (*I_0_*) | Ratio  (*I* / *I_0_*) | Texture Coefficient  (*T_c_*) |
| --- | --- | --- | --- | --- | --- |
| ZnO@CF-0.1 | (100)  (002)  (101) | 0.54  0.82  1 | 0.57  0.44  1 | 0.95  1.86  1 | 0.74  1.47  0.79 |
| ZnO@CF-0.2 | (100)  (002)  (101) | 0.66  0.64  1 | 0.57  0.44  1 | 1.17  1.45  1 | 0.97  **1.21**  0.83 |
| ZnO@CF-0.3 | (100)  (002)  (101) | 0.64  0.67  1 | 0.57  0.44  1 | 1.12  1.51  1 | 0.93  **1.25**  0.83 |
| ZnO@CF-0.4 | (100)  (002)  (101) | 0.54  1  0.93 | 0.57  0.44  1 | 0.94  2.27  0.93 | 0.68  **1.65**  0.67 |
| ZnO@CF-0.5 | (100)  (002)  (101) | 0.36  1  0.63 | 0.57  0.44  1 | 0.63  2.27  0.63 | 0.54  **1.93**  0.53 |

*Standard intensities (*I_0_*) were obtained from JCPDS No. 00-036-1451

The texture coefficient for each crystal plane (hkl) was calculated using the Harris formula:

$$T_{c}\left( hkl \right)= \frac{I(hkl)/I_{0}(hkl)}{\frac{1}{n}\sum_{i=1}^{n} [I(h_{i}k_{i}l_{i})/I_{0}(h_{i}k_{i}l_{i})]}$$

Where $I(hkl)$ is measured relative intensity of the (hkl) plane from the XRD patterns, $I_{0}(hkl)$ is standard intensity of the (hkl) plane for bulk ZnO (JCPDS card No. 00-036-1451), and n is the number of diffraction planes considered in the analysis (n=3 for (100), (002), and (101) planes).

**
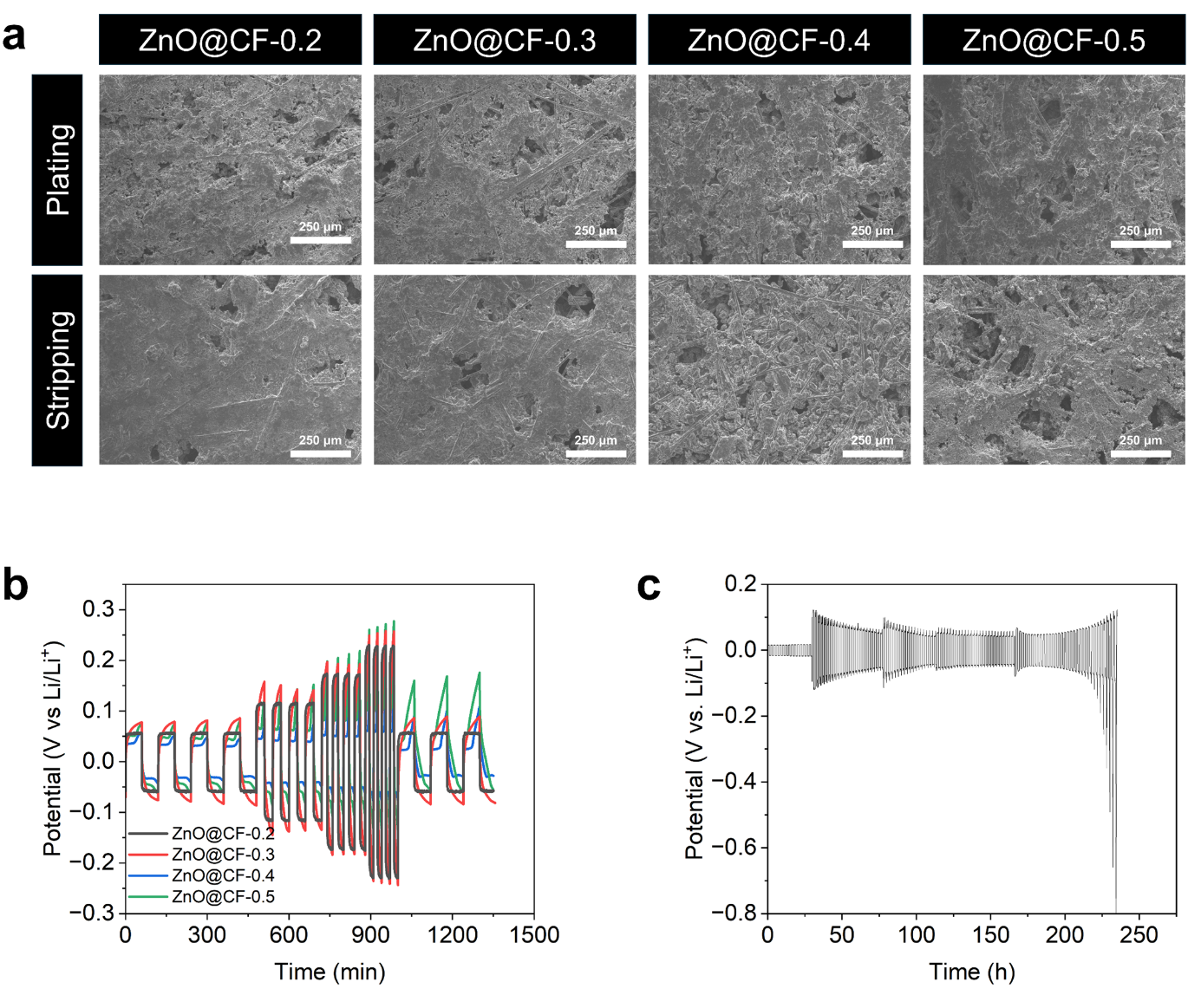
 Figure S7.** Interfacial stability and kinetic characterization of facet-engineered ZnO@CF electrodes according to ETW conditions. (a) Top-view SEM images of ZnO@CF-0.2, -0.3, -0.4, and -0.5 electrodes after the initial Li plating and the 10th stripping cycle, illustrating the facet-dependent morphological evolution. (b) Rate capability of symmetric cells using various ZnO@CF electrodes at stepwise increasing current densities (1, 2, 3, and 4 mA cm⁻²) and subsequent recovery to 1 mA cm⁻² (1 mAh cm⁻² per cycle). (c) Long-term voltage-time profile of a bare Li|Li symmetric cell, serving as a baseline for comparing interfacial stability.


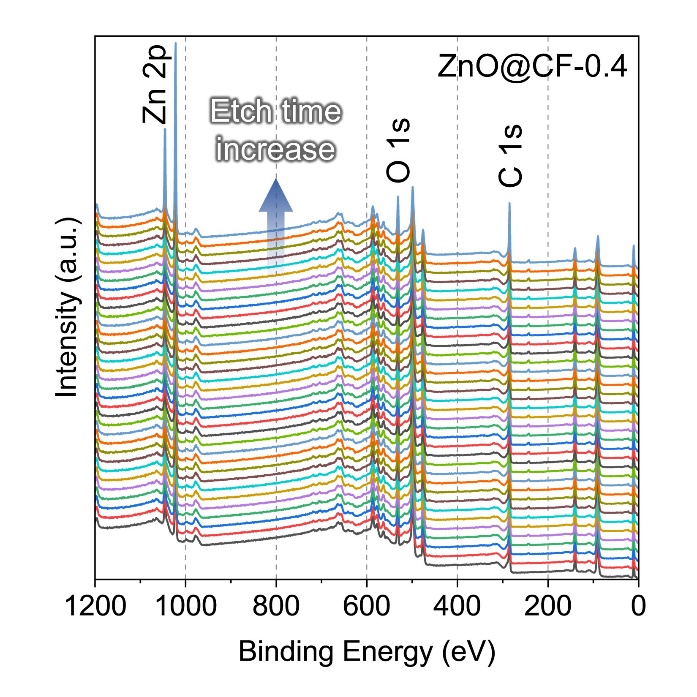


**Figure S8.** XPS depth-profile survey spectra of ZnO@CF-0.4 obtained during sequential Ar^+^ etching (35 cycles, 10 s per cycle; total 350 s) at an ion energy of 2000 eV, showing the evolution of the Zn 2p, O 1s, and C 1s signals with increasing etch depth.


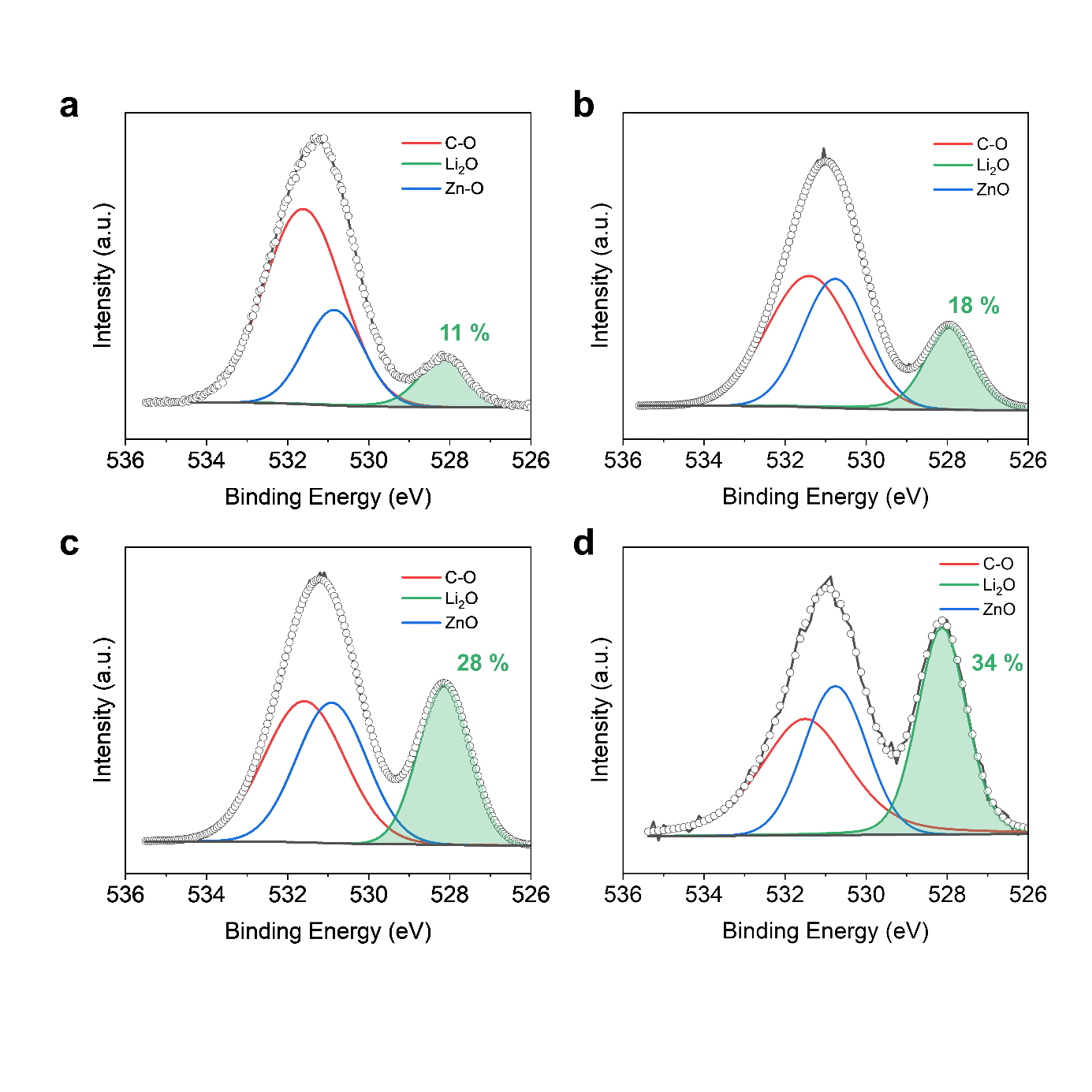


**Figure S9.** XPS O 1s spectra of ZnO@CF composites at 0 V vs. Li/Li^+^. (a) ZnO@CF-0.2, (b) -0.3, (c) -0.4, and (d) -0.5, with deconvoluted peaks assigned to C–O, Li_2_O, and Zn–O. The green values indicate the relative atomic percentage of Li_2_O, calculated based on the peak area ratio within the total O 1s bonding.


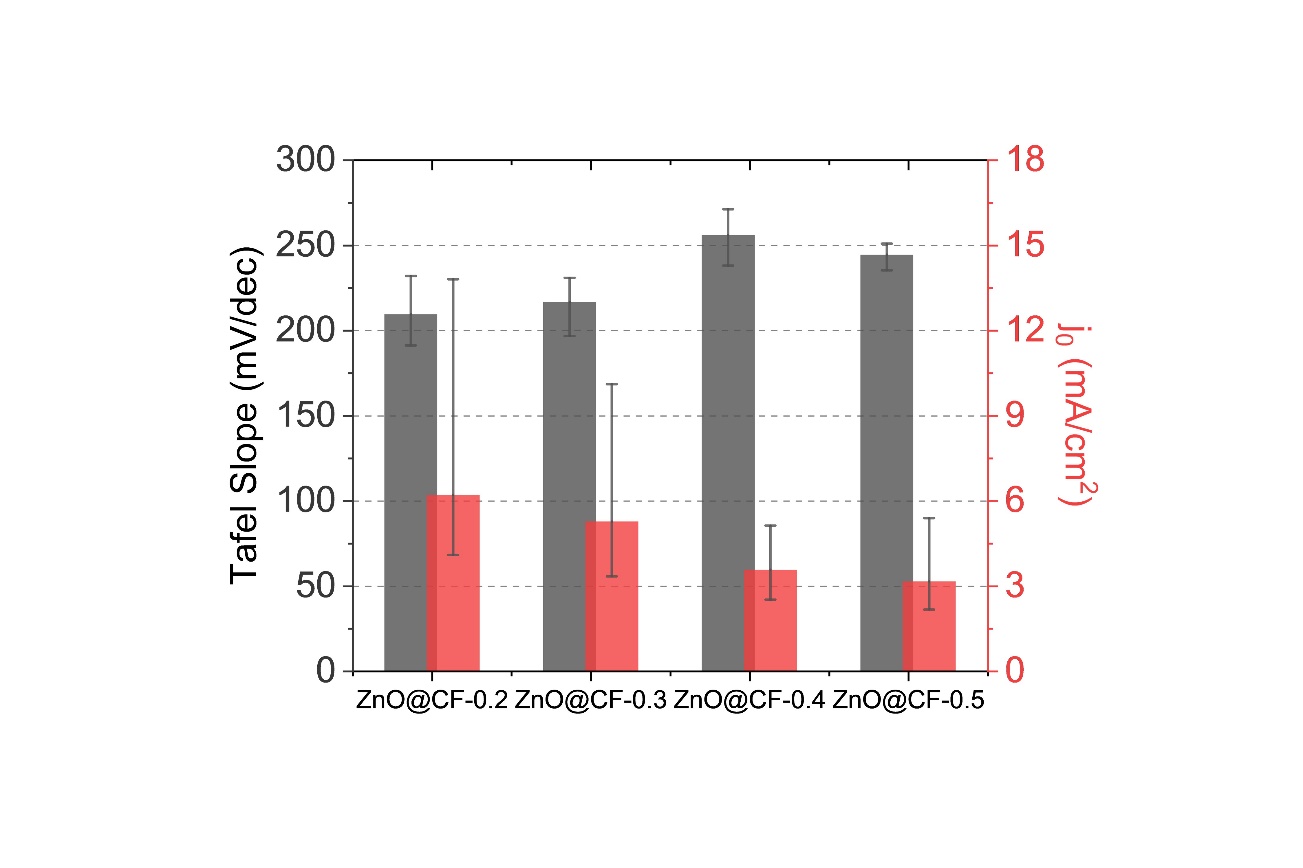


**Figure S10.** Average Tafel slopes (black bars, left axis) and exchange current densities (*j_0_*, red bars, right axis) obtained from linear fitting of the Tafel plots. The data represent the mean of five measurements (*n=5*), and the error bars indicate the minimum–maximum range.

**Table S4.** Average value of Tafel slope (*β*) and exchange current density (*j_0_*) of the ZnO@CF electrodes.

|  | **ZnO@CF-0.2** | **ZnO@CF-0.3** | **ZnO@CF-0.4** | **ZnO@CF-0.5** |
| --- | --- | --- | --- | --- |
| **β (mV/dec)** | 209.5 | 216.7 | 256.1 | 244.5 |
| **j_0_ (mA/cm^2^)** | 6.2 | 5.26 | 3.56 | 3.15 |

**
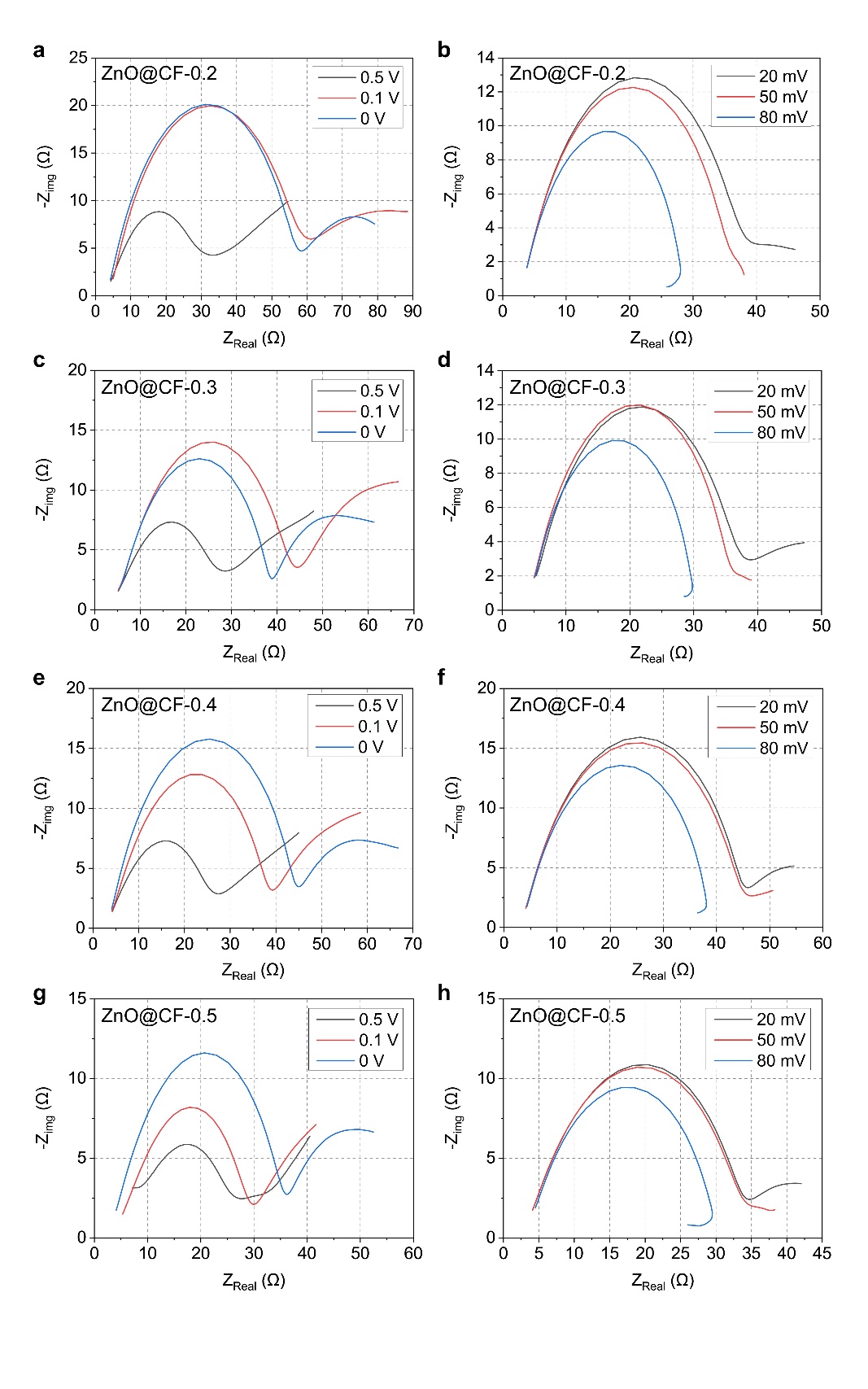
**

**Figure S11.** Nyquist plots of ZnO@CF electrodes under Li-ion-battery (LIB)-type and Li-metal-battery (LMB)-type conditions. Electrochemical impedance spectroscopy (EIS) spectra at 0, 0.1, and 0.5 V vs. Li/Li^+^ for (a) ZnO@CF-0.2, (c) ZnO@CF-0.3, (e) ZnO@CF-0.4, and (g) ZnO@CF-0.5. Potentio electrochemical impedance spectroscopy (PEIS) spectra under direct-current (DC) biases of 20, 50, and 80 mV for (b) ZnO@CF-0.2, (d) ZnO@CF-0.3, (f) ZnO@CF-0.4, and (h) ZnO@CF-0.5.

**
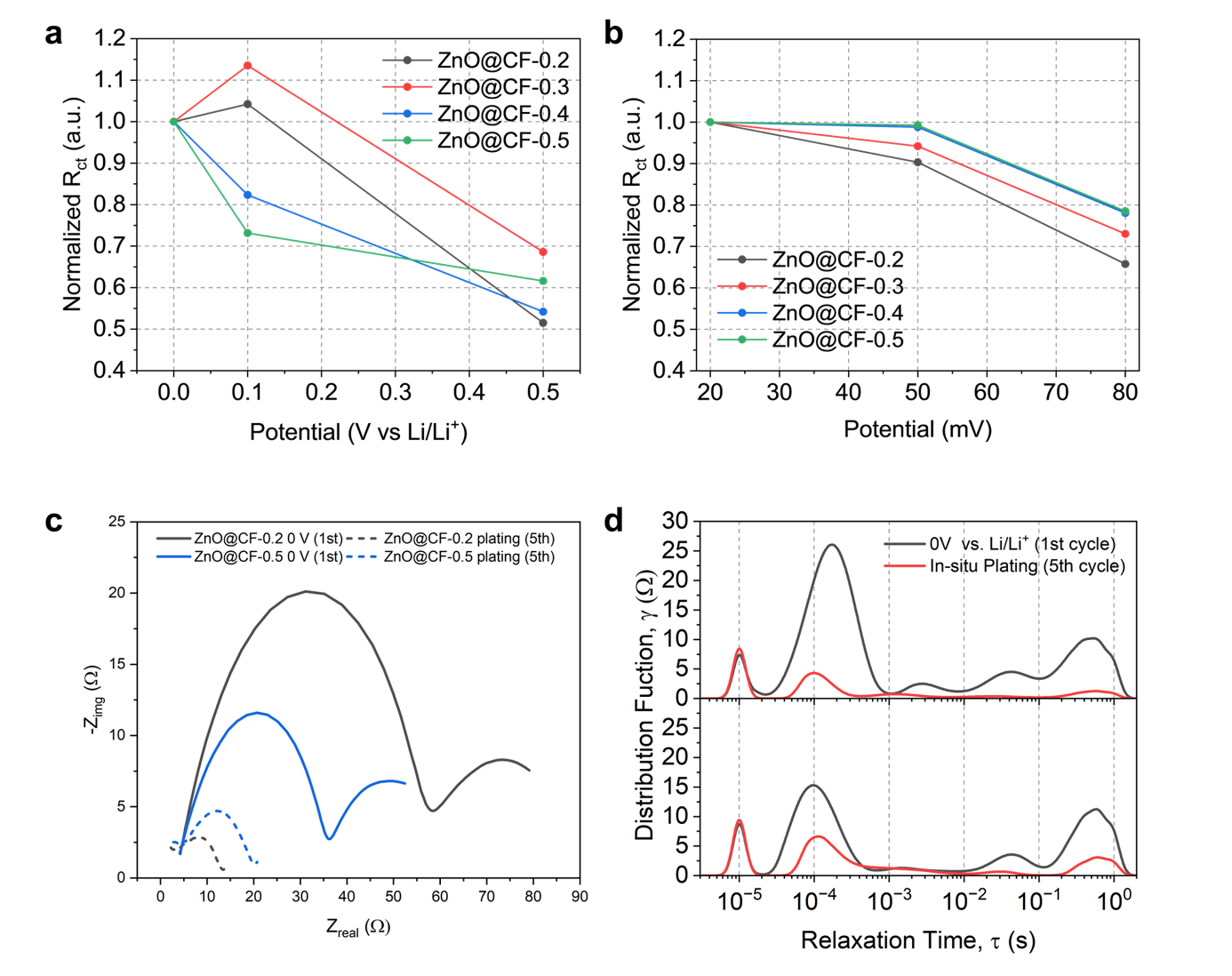
**

**Figure S12.** Electrochemical kinetic and interfacial evolution analysis via EIS and DRT. (a) Normalized R_ct_ trends measured under LIB–type conditions (no bias), showing the resistance transition during the intercalation and conversion process. (b) R_ct_ values obtained from PEIS under LMB–type conditions with a DC bias applied at 0 V, illustrating the facet-dependent onset of Li plating. (c) Nyquist plots comparing the interfacial impedance of the optimized ZnO@CF-0.2 (black) and the (002)-dominant ZnO@CF-0.5 (blue) at the initial state (solid lines) and during in-situ Li plating at the 5th cycle (dashed lines). (d) Distribution of relaxation times (DRT) analysis derived from the EIS spectra in (c) for ZnO@CF-0.2 (top) and ZnO@CF-0.5 (bottom). The profiles demonstrate the evolution of characteristic resistance peaks from the initial 0 V vs. Li/Li⁺ state (black) to the dynamic Li-plating state at the 5th cycle (red), illustrating the superior interfacial stability and faster charge-transfer kinetics of the (101)-dominant ZnO@CF-0.2 electrode.


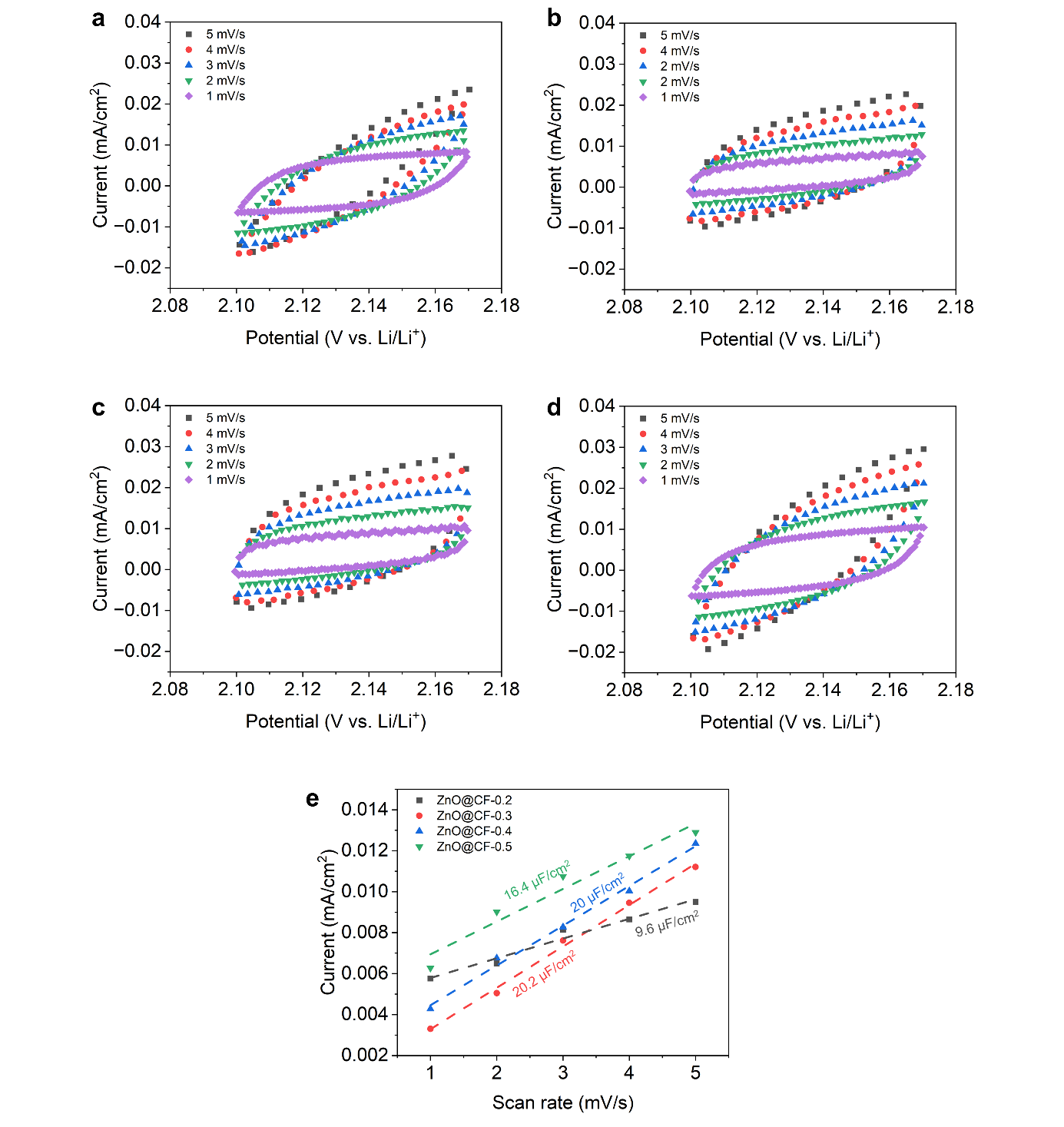


**Figure S13.** Electrochemical active surface area (ECSA) measurements of the electrodes. Cyclic voltammetry (CV) curves of (a) ZnO@CF-0.2, (b) ZnO@CF-0.3, (c) ZnO@CF-0.4, and (d) ZnO@CF-0.5 at various scan rates from 1 to 5 mV s⁻¹ in the non-Faradaic potential range (2.10–2.17 V vs. Li/Li⁺). (e) Linear fitting of the capacitive current density (Δ*j* = (*j*_anodic_ − *j*_cathodic_)/2) at 2.135 V (vs. Li/Li⁺) as a function of the scan rate. The slope represents the double-layer capacitance (Cdl), which is proportional to the ECSA.


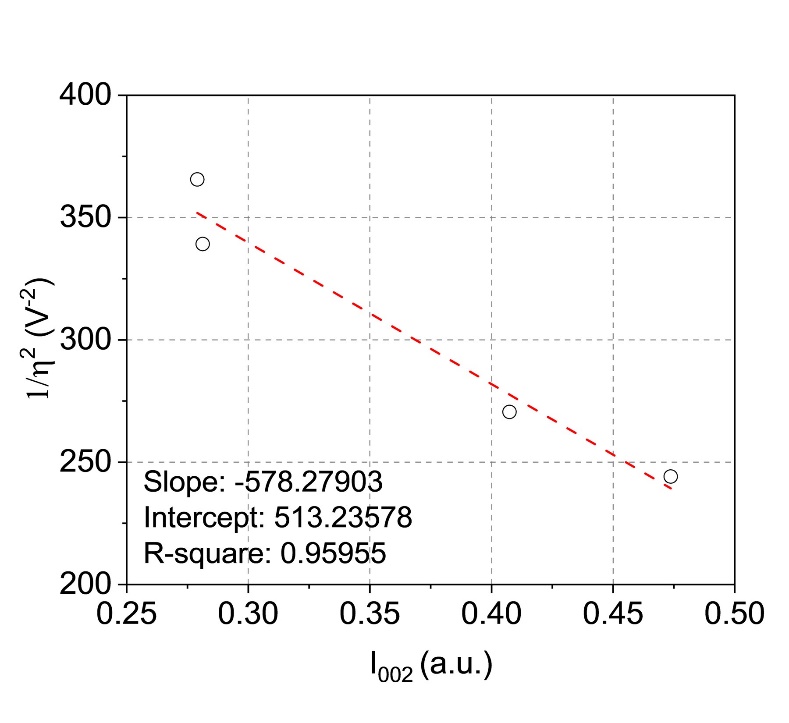


**Figure S14.** Linear correlation between the (002) peak intensity (*I_002_*) and the inverse square of the overpotential (*1/η^2^*), highlighting the facet-dependent nucleation behavior of ZnO.
